# Population dynamics and habitat sharing of natural populations of *Caenorhabditis elegans *and *C. briggsae*

**DOI:** 10.1186/1741-7007-10-59

**Published:** 2012-06-25

**Authors:** Marie-Anne Félix, Fabien Duveau

**Affiliations:** 1 Institut Jacques Monod, CNRS - University of Paris VII, 15 rue H. Brion, 75205 Paris cedex 13, France; 2 Institut de Biologie de l'Ecole Normale Supérieure, CNRS - ENS - Inserm, 46 rue d'Ulm, 75230 Paris cedex 05, France

## Abstract

**Background:**

The nematode *Caenorhabditis elegans *is a major model organism in laboratory biology. Very little is known, however, about its ecology, including where it proliferates. In the past, *C. elegans *was mainly isolated from human-made compost heaps, where it was overwhelmingly found in the non-feeding dauer diapause stage.

**Results:**

*C. elegans *and *C. briggsae *were found in large, proliferating populations in rotting plant material (fruits and stems) in several locations in mainland France. Both species were found to co-occur in samples isolated from a given plant species. Population counts spanned a range from one to more than 10,000 *Caenorhabditis *individuals on a single fruit or stem. Some populations with an intermediate census size (10 to 1,000) contained no dauer larvae at all, whereas larger populations always included some larvae in the pre-dauer or dauer stages. We report on associated micro-organisms, including pathogens. We systematically sampled a spatio-temporally structured set of rotting apples in an apple orchard in Orsay over four years. *C. elegans *and *C. briggsae *were abundantly found every year, but their temporal distributions did not coincide. *C. briggsae *was found alone in summer, whereas both species co-occurred in early fall and *C. elegans *was found alone in late fall. Competition experiments in the laboratory at different temperatures show that *C. briggsae *out-competes *C. elegans *at high temperatures, whereas *C. elegans *out-competes *C. briggsae *at lower temperatures.

**Conclusions:**

*C. elegans *and *C. briggsae *proliferate in the same rotting vegetal substrates. In contrast to previous surveys of populations in compost heaps, we found fully proliferating populations with no dauer larvae. The temporal sharing of the habitat by the two species coincides with their temperature preference in the laboratory, with *C. briggsae *populations growing faster than *C. elegans *at higher temperatures, and *vice *at lower temperatures.

## Background

The nematode *Caenorhabditis elegans *is a top model organism in biology. The wealth of data on its biology has greatly contributed to advancing knowledge in developmental, cellular and molecular biology. Yet the laboratory biology of *C. elegans *is disconnected from its natural context, and studies of natural populations are in their infancy [1]. One aim in deciphering the natural history of *C. elegans *is to provide an evolutionary and ecological context for the accumulated genomic and mechanistic knowledge. A complementary aim is to develop *C. elegans *as a model species for evolutionary and ecological studies, making use of genetic and molecular tools and knowledge that this organism affords, and integrating the two facets of its biology. In addition to the experimental advantages of *C. elegans*, its mode of reproduction with selfing hermaphrodites and facultative outcrossing to males makes it a favorable system to study the regulation of outcrossing and its evolutionary consequences. Recent years have thus seen a surge in interest for using *C. elegans *in evolutionary studies [1-4]. Yet, even the habitat where it feeds and reproduces has so far not been well characterized.

*C. elegans *develops in three to four days at 20°C in the laboratory, going through a short embryonic development and four juvenile/larval stages separated by molts, followed by the reproductive adult [5]. In conditions of low food, crowding and high temperature, the young larvae of *C. elegans *develop into an alternative L3 diapause stage called the dauer. Specification of dauer development occurs at the end of the L1 stage, which then molts into an L2d pre-dauer stage. Entry into the dauer stage is still reversible at this stage if better conditions resume. If not, the L2d larva molts into a dauer larva, which can pause in its development for several months, without feeding, and is resistant to a range of stresses. If the dauer larva encounters a new favorable environment, it will start feeding, develop into post-dauer L4 and adult stages.

Previously, *C. elegans *has been sampled mostly in compost heaps [6-9] where individuals were found predominantly in the dauer stage [7,9] and also found in association with invertebrates, such as snails and isopods [7,9,10]. We occasionally found *C. elegans *in rotting fruits [9], which drove us to a more systematic survey of rotting vegetal material on different continents and islands. We and others thereby found many new *Caenorhabditis *species, most of them in tropical regions [11]. A genomic analysis was conducted on a worldwide set of *C. elegans *isolates and suggested the recent occurrence of several worldwide selective sweeps affecting large genomic regions [12].

Understanding the habitat, food, and pathogens that *C. elegans *experiences may provide clues to the possible selective pressures that it encounters in the wild, which may have driven these intense selective sweeps.

Here we describe the systematic local sampling of *C. elegans *and *C. briggsae *in rotting fruits and stems. *C. elegans *and *C. briggsae *are the two most common *Caenorhabditis *species in mainland France. The approximate census size and frequency of developmental stages could be assessed by analyzing fresh samples that were examined in the laboratory within a few hours after collection. Rotting fruits in orchards and rotting herbaceous stems in wood and shrubland areas, support large proliferating populations, some of them without any diapausing dauer larvae. Remarkably, *C. elegans *and *C. briggsae *often co-exist in the same locations and substrates. In order to study their relative abundance and distribution, we systematically sampled a large spatio-temporal structured set over four years in an apple orchard in Orsay and over three years in a wood in Santeuil. The two species present a strikingly different temporal distribution during the year, with *C. briggsae *dominating in summer and *C. elegans *appearing during the fall, as *C. briggsae *gradually fades out. This seasonal shift correlates with their respective temperature preferences in the laboratory, as shown in a laboratory competition experiment.

## Results

### *C. elegans *and *C. briggsae *proliferate in the same natural habitats in rotting fruits and stems and are found associated with the same invertebrates

**Figure 1:**
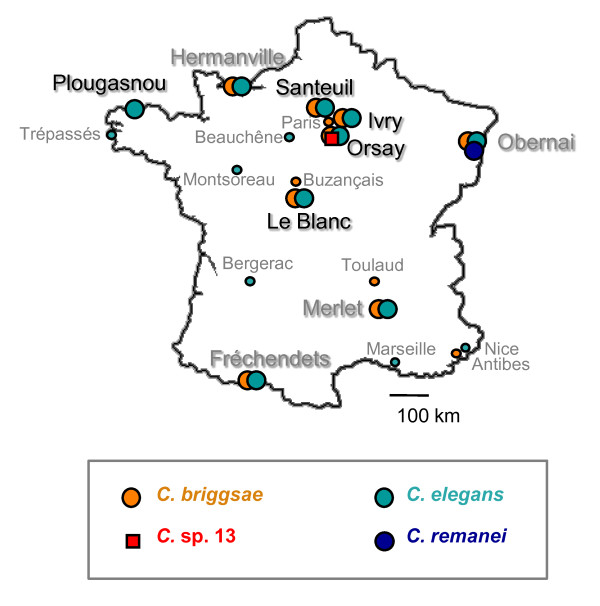
**Sampling locations in France**. Both *C. elegans *and *C. briggsae *were found in the locations where extensive sampling was performed (large disks), except in the Western France (Plougasnou) location. Locations with names in black are those in Table 1. Others are from [9,52] or new locations that have been only occasionally and sparsely sampled (small symbols and fonts) (Credits: Bergerac: Victor Nigon [53]; Buzançais: Jean-Baptiste Pénigault; Marseille, Nice, Antibes: Christian Braendle).

**Figure 2:**
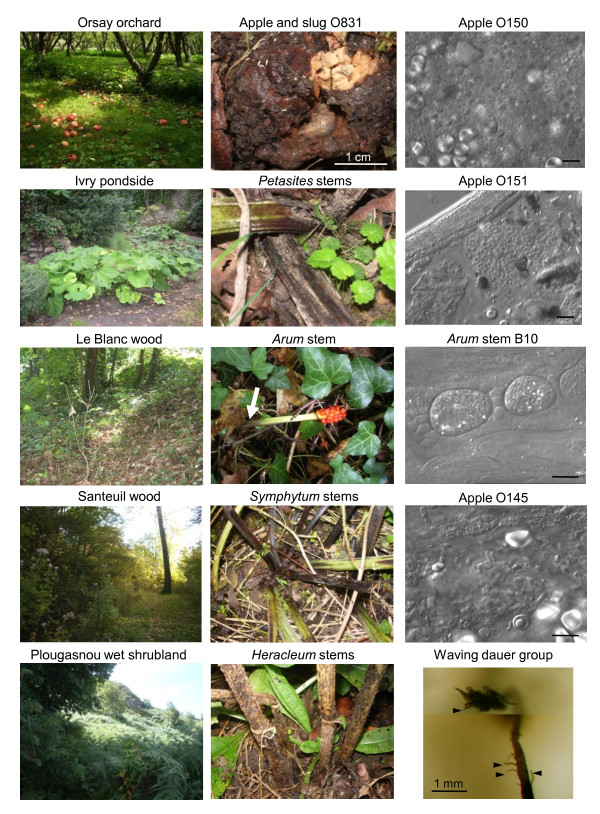
**Landscapes and substrates with proliferating *Caenorhabditis *populations**. The left panels depict landscapes in mainland France locations, as referenced in Table 1 and shown on the map on Figure 1. The central panels show examples of the corresponding sampled substrates that yielded *Caenorhabditis*. The right panels, except the bottom-most one, are Nomarski pictures of samples, showing a very heterogeneous composition, with bacteria, fungi and unidentified structures. A young *Caenorhabditis *juvenile is visible in the second panel from the top, labeled "Apple O151". The bottom panel is a juxtaposition of two focal planes of a large waving group of thousands of dauers, standing on rotten apple O673 (at the bottom of the picture). A few individual dauer larvae are indicated by arrowheads. The corresponding movie is available on request. Bars: 10 μm, unless otherwise indicated. On the *Arum *panel, the white arrow designates the rotting part of the stem. On the upperleft of the apple O831 panel, the small white animals are springtails.

Rotten vegetal habitats were sampled in different locations in France (Figure 1) and assayed for the presence, abundance and developmental stage distribution of *Caenorhabditis *nematodes. *Caenorhabditis *populations were found on various substrates, which include rotting fruits and rotting stems (Table 1 and illustrated in Figure 2). By exploring these habitats, we found for the first time *Caenorhabditis *populations that were in proliferative stages, without any dauer juveniles (Table 2 Additional Files 1 and 2).

**Table 1:** Habitats of *C. elegans *and *C. briggsae *in temperate regions of France

Location name and landscape type	Coordinates (latit., longit.)	Substrates (proportion of *Caenorhabditis *positive)	Species
Orsay orchard	48.702, 2.172	Apples, pears; rhubarb stems	*Cel + Cbr*

Orsay pond side	48.701, 2.180	*Petasites *stems	*Cel + Cbr*

Ivry-sur-Seine city garden with pond	48.809, 2.386	*Petasites *stems (68%, n = 28); snails, slugs	*Cel + Cbr*

Le Blanc garden	46.629, 1.059	Compost; fruits (apples, pears, plums, tomatoes, peaches); snails	*Cel + Cbr*

Le Blanc wood	46.639, 1.051	*Arum *stems, acorns, walnut skins	*Cel*

Santeuil orchards	49.126, 1.962	Apples, grapes; slugs, snails	*Cel + Cbr*

Santeuil wood along small stream	49.121, 1.951	*Petasites*, *Heracleum*, *Symphytum*, *etc*. stems; snails	*Cel + Cbr*

Plougasnou-Primel garden	48.709, -3.813	Compost; fruits; snails; *Arum *stems	*Cel*

Plougasnou coastal heath	48.705, -3,795	*Arum*, *Heracleum*, *Pteridium *stems; *Tamus *leaves/stem; sloe fruits	*Cel*

This table includes the locations that were repeatedly sampled over several years. *Cbr*, *C. briggsae*; *Ce*, *C. elegans; *latit., latitude; longit., longitude; n, number.

**Table 2:** Stage composition of *Caenorhabditis *populations

Sample ID	Substrate type	Substrate ID	Sample weight (total)	Population in plated sample	Total census (Log)	Population stage?	Species
O661	fruit	Rosaceae - *Malus domestica*	52 (94)	~ 30, all stages	2	P1	Cbr (9/9)
O662	fruit	Rosaceae - *Malus domestica*	25 (39)	~ 500, 20 ad, 300 d, 100 L2d, 100 L1	3	P3	Cbr (20/20)
O663	fruit	Rosaceae - *Malus domestica*	15 (23)	~ 20 (d?)	2	?	Cbr (12/12)
O664	fruit	Rosaceae - *Malus domestica*	10 (20)	~ 10,000, 250 ad, 7000 d, 3000 L2d	5	P3	Cbr (17/17)
O665	fruit	Rosaceae - *Malus domestica*	19 (32)	~ 2,000, 60 ad, 600 d, 600 L2d, 600 L1	4	P3	Cbr (18/18)
O666	fruit	Rosaceae - *Malus domestica*	27 (44)	~ 30 (d?)	2	?	Cbr (5/5)
O667	fruit	Rosaceae - *Malus domestica*	46 (83)	2 (1 ad, 1 L2d)	1	?	Cbr (2/2)
O668	fruit	Rosaceae - *Malus domestica*	26 (43)	~ 500-, 40 ad, 20 L4, 20 L3, 60 d, 200 L2, 100 L1	3	P2	Cbr (18/18)
O669	fruit	Rosaceae - *Malus domestica*	22 (37)	~ 1000+, 100 ad, 100 L4, 100 L3, 500 L2d, 500 L1,10 d	4	P2	Cbr (20/20)
O670	fruit	Rosaceae - *Malus domestica*	36 (62)	1	1	?	Cbr (1/1)
S101	stem	Apiaceae - *Heracleum sphondylium*	6	~ 30, dauers, 1 L3, all stages	2	P	Cel (6/6)
S102	stem	Asteraceae	4	~ 30, non-dauer larvae	2	P1	Cel (6/6)
S103	stem	Asteraceae - *Cirsium oleraceum?*	8	~ 2000 total, proportions ca. 30 ad, 200 d, 100 L2d, 100 L1	4	P3	Cel (6/6)
S104	stem	Asteraceae - *Petasites *sp.	1	2 dauers (L4 next day)	1	D	Cel (3/3)
S105	stem	Asteraceae - *Cirsium oleraceum?*	5	~ 1000 total, proportions ca. 5 ad, 10 L4, 5 L3, 50 L2d, 50 L1	3	P2	Cel (6/6)
S106	stem	Apiaceae - *Heracleum sphondylium*	1	~ 500 total, proportions ca. 30 ad, 15 L4, 10 L3, 30 L2, 30 L1	3	P1	Cel (6/6)
S107	stem	Asteraceae - *Petasites *sp.	4	6, stage unclear	1	?	Cel (6/6)
S108	3 snails	3 snails		-	0	-	-
S109	stem	Asteraceae - *Cirsium oleraceum?*	2	few, at least 1 dauer, next day 1 L2	1	?	Cel (5/5)
S110	stem	Asteraceae - *Petasites *sp.	6	~ 50, 5 ad, 5 L4, 5 L3, 5 d ?	2	P	Cel (6/6)

Scoring of *Caenorhabditis *populations in ten Orsay apples (O661-670) and ten Santeuil samples (S101-S110). These sets were chosen to represent a variety of *Caenorhabditis *population size and type, and maximize the proportion of positive samples (that is, the presence of *Caenorhabditis *is less frequent than would be suggested by these sets; see Additional Files 1 and 2 and Figure 5). Only a part of each apple was analyzed, which is taken into account for in the total census. Weight is in grams. Total census is on a Log scale. d: dauer larva. ad: adult. The tentative stage of the population is indicated: D, dauer stage; P, proliferating; P1, early stage of proliferation (no dauers); P2, intermediate stage (L3 and L4 stages present, as well as L2d or dauers); P3, late stage (many L1, L2d, dauers). The *Caenorhabditis *species was identified for a set of individuals of each population, as indicated in the last column. ~, approximately; Cbr: *C. briggsae; *Cel: *C. elegans*.

*C. briggsae *and *C. elegans *were both found on rotting fruits of the same species, such as apples, pears, plums, peaches, tomatoes and figs. A mix of both species could be found proliferating in the very same individual fruit. Plant species in the fruits of which we found only one of the *Caenorhabditis *species, but not the other (likely because of undersampling of these plant species, or seasonal patterns; see below) were *C. briggsae *on wild cherries and peaches in Gif-sur-Yvette and Le Blanc, respectively, and *C. elegans *once on wild sloe fruits in Plougasnou. Quantitative data on *Caenorhabditis *occurrence in several samples at the same location are found in Additional Files 1 and 2. We focused on sampling rotting apples in the Orsay orchard [see Additional File 1]. Summing over the four years of sampling in Orsay, we found *C. elegans *and *C. briggsae *each at a frequency of around 20% of the rotten apples (n = 429; but see below for the heterogeneity among timepoints). Analysis of rotten fruits still hanging on the trees (Orsay, 6 and 14 October 2008, apples O70-77 and O98-O105; [see Additional File 1] rather than fallen on the ground under the same tree did not yield any *Caenorhabditis*, but did yield other nematodes such as fungi-eating aphelenchoides.

Proliferating *Caenorhabditis *populations were also found in a variety of rotting stems of herbaceous plants: *Petasites*, *Heracleum*, *Arum*, *Symphytum *species, and so on (Figure 2 and Table 1). Several of these are bi-annual plants with relatively thick stems that rot in summer or fall off during the second year of growth. Again, both *C. briggsae *and *C. elegans *were found proliferating in stems of the same species, and sometimes in the same individual stem. We focused on the Santeuil wood for rotting stems and sampled over three years in October [see Additional File 2 sheet 1]. Each year, *C. elegans *was found in Santeuil on about 50% of the rotten stems and *C. briggsae *on about 10% of the rotten stems (n = 88 in total). Note however that these proportions are only indicated as an example of a habitat and location where *Caenorhabditis *was abundant and that they may vary as a function of season and location [see Additional File 2].

Substrates negative for *Caenorhabditis*, but not for other nematodes, include rotting wood, decomposing grass and generally rotting leaves. *Caenorhabditis *can be only occasionally found in soil, for example immediately below a rotting apple [See Additional File 1, 14 October 2008] or a rotting stem [see Additional File 2, S174]. In contrast, *Oscheius tipulae *is very commonly found in soil in the dauer stage [13], and was here found for the first time in proliferative stages in rotting fruits and stems, like *Caenorhabditis *species.

Other nematodes could be commonly found proliferating in rotting fruits and stems, sometimes in large populations: i) several species of the *Eurhabditis *clade [14] including *O. tipulae *and *O*. sp. 2, species with tube-waving dauers (resembling *Rhabditis *sp. SB347 [15]), and several others; ii) *Pristionchus *spp.; iii) *Panagrellus *(fruits only) and *Panagrolaimus *(both fruits and stems) spp.; iv) *Rhabditophanes *sp. (cold seasons); v) *Mesorhabditis *sp.; and vi) fungi-eating aphelenchoides (fruits).

Besides nematodes, the biotic environment includes bacteria and phages, fungi, acellular and cellular slime molds, ciliates, slugs and snails, collembola, mites, insect larvae (for example, often *Drosophila *spp. in rotting fruits, but not in stems), isopods, myriapods, and so on. At a small scale, when observed under the high-power light microscope, the rotting fruits and stems are colonized by an assemblage of bacteria and fungi, the latter under both their yeast and hyphal forms. These microbes form growing colonies that may be separated by remains of plant walls and thus constitute heterogeneous micro-environments (Figure 2, right panels).

Possible invertebrate carriers of nematodes were also sampled and analyzed for the presence of nematode species. *C. elegans *and *C. briggsae *were found on the same species of arthropod or mollusk hosts, often in the same local host population or even the same individual host. Specifically, as previously reported for *C. elegans *[9,10,16,17], we found *C. elegans *and *C. briggsae *on diverse isopods, millipedes, snails and slugs [see Additional Files 1 and 2]. We also found once *C. briggsae *on a dead adult male firefly in Gif-sur-Yvette and on insects (insect larva and small coleopter) associated with a rotten apple with a large *C. briggsae *population (Orsay O634, 26 July 2010), and *C. elegans *on the surface of an annelid in a rotten apple with a large *C. elegans *population (Orsay 0843, 11 November 2011). Whether there is any higher specificity of association other than co-occurrence of nematode and host in the same rotting material is unclear. Systematic sampling of the diverse set of potential invertebrate carriers outside rotting fruits/stems still remains to be performed to determine whether insects, myriapods and annelids carry dauer larvae beyond one patch of rotting habitat. *Drosophila *species share the rotting fruit substrate with *Caenorhabditis*. However, catching *Drosophila *adults never yielded *Caenorhabditis*, but did yield *Panagrellus *sp., both in Le Blanc and Orsay (M-AF and T. Bélicard). In Le Blanc, *Drosophila *flies were caught at a given spot of the vegetable garden spot with mouth aspiration tubes (courtesy of D. Anxolabéhère) or baits of vinegar-coated fly tubes and worm agar plates and then plated individually or by groups of five flies. *Panagrellus *sp. was isolated in four out of six five-fly sets and in two out of seven individual flies, suggesting a proportion of about 20% of the flies being carriers.

The *Caenorhabditis *developmental stage previously shown to be carried by isopods and terrestrial mollusks was the dauer larva for *C. elegans *and related species such as *C. remanei *[7,10,18]. We have observed dauer larvae being discharged from isopods [9]. Yet, so far we had not reported the developmental stage of *Caenorhabditis *individuals on mollusks, because we could only observe them on the plate several days after sampling. Here we succeeded in observing nematodes within hours of sampling, by better shearing and spreading the snail or slug tissues on large plates, thus allowing the worms to exit more easily. Surprisingly, *C. elegans *and *C. briggsae *were not restricted to the dauer stage but occurred as adults and mixed larval stages, indicating the presence of proliferating populations [see Additional File 2, Ivry and Santeuil S162A]. Although we do not know in which mollusk body part the nematodes were harbored, it is possible that the *Caenorhabditis *individuals were inside the intestinal lumen rather than in the host body. In one instance (slug O831bis), we could observe that dauer juveniles were found both in intestinal contents and under the mantle.

### Populations differ by their developmental stage composition

Most of the populations were analyzed on the day of sampling, which enabled us to determine the population census and developmental stage of individual worms. The corresponding data are in Additional File 1 for Orsay and in Additional File 2 for the other locations. Table 2 shows examples of population scoring on ten Orsay apples (O661-670) with *C. briggsae *and ten Santeuil samples (S101-S110) with *C. elegans*.

Population sizes in a given sample spanned one to more than 10,000 *Caenorhabditis *individuals, and were expressed on a Log scale with an index of 1 for 1 to 10 individuals, 2 for 11 to 100, 3 for 10^2 ^to 10^3^, 4 for 10^3 ^to 10^4^, 5 for > 10^4^. Some populations of small census size (index 1 or 2) contained individuals that were all in dauer, corresponding to non-proliferating populations in migrating or pausing stage. Some populations of moderate census (index 2 or 3) included all stages that would be expected from a proliferating population (adults and larval stages with no L2d or dauer stages, labeled P1 in Table 2). Samples with high census (index 4-5) always contained some L2d and dauer larvae (labeled P2), and some did not contain L3 and L4s at all (labeled P3), indicative of populations entering the dauer stage at the end of a proliferative stage. Note that some populations of smaller size were also found to include dauer and L2d larvae. This perhaps occurs when the environment is only able to sustain a smaller population and entry into dauer occurs at a lower abundance, or when most animals have already migrated out and/or when only some immigrating dauers have resumed development.

We could not detect any difference between *C. briggsae *and *C. elegans*, nor between fruit and stem substrates, as to the general composition of populations or the overall density of *Caenorhabditis *in the sample [see Additional Files 1 and 2]. In the specific samples where both species were found together, their populations were composed of similar developmental stages (data not shown). The only notable exception is apple O738 on 8 November 2010, where the only dauer out of about 50 animals was *C. briggsae*, the rest being *C. elegans*. At this late time in the season (see below), most *C. briggsae *were found in the dauer stage.

Dauers can be seen waving individually or in groups at the surface of any pointed structure of the habitat. This nictation behavior is thought to help in finding an invertebrate host for migration [17,19]. We observed that the masses of dauer larvae can reach centimeter-size, with thousands of dauers oscillating en masse (Figure 2, bottom right panel). This behavior appears similar in *C. elegans *and *C. briggsae*.

### Sex, food and infections

The isolation of *Caenorhabditis *individuals from fresh samples provides information about their life in the wild, especially concerning mating, food and pathogens.

*C. elegans *and *C. briggsae *both reproduce through selfing XX hermaphrodites and facultative X0 males. In agreement with previous reports [7,9], males were rarely observed in natural populations. We occasionally observed single adult males in large populations, sometimes a few [see Additional File 2, Le Blanc B11-31 with *C. briggsae*], which could be the result of spontaneous X-chromosome non-disjunction. When we isolated L1-L3/dauer individuals from the population to determine their species, without knowing their sex [see Additional Files 1 and 2], we never isolated a male. An exception is the stem sample population P141 where we found two males out of 15 isolated *C. elegans *individuals (both in the dauer stage; there were no adults in this population) [see Additional File 2]. Mating of a male with a hermaphrodite results in half of the cross-progeny being male. By isolating hermaphrodite adults from the wild, it is thus possible to assess whether they were mated by scoring the male frequency in their progeny. Out of hundreds of isolated hermaphrodite adults of each species, we observed a F1 brood with a large proportion of males in one of 13 individual adults isolated from apple O111 for *C. elegans *and one in five adults in apple O170 for *C. briggsae *[see Additional File 1] (in stem S210, males were found in the progeny of an adult isolated four days after sampling, which renders unclear whether it had been mated in the wild). We thus confirm that outcrossing does occur, albeit very infrequently in natural populations of *C. elegans *and we extend here the observation to *C. briggsae*.

**Figure 3:**
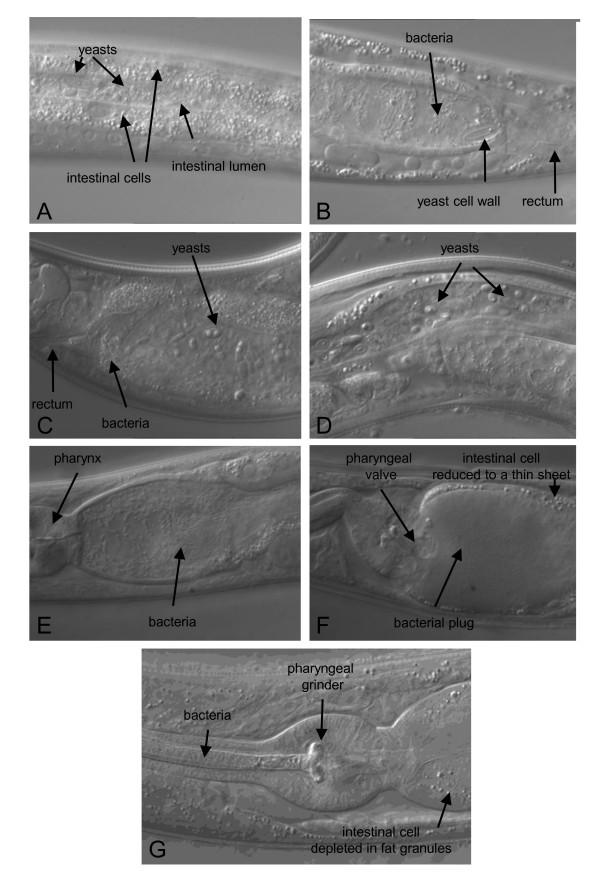
**Alimentary tracts of freshly sampled animals**. (**A**) *C. elegans *late L3 larva, freshly isolated from a rotting apple (#7) in Santeuil, with bacteria and yeast in the intestinal lumen. (**B**) *C. elegans *adult in the same sample, with empty fungal cell walls in the intestinal lumen. (**C**). *C. elegans *adult freshly isolated from a rotting apple (O145) from Orsay. (**D**) *C. briggsae *adult in a rotting peach from Le Blanc. (**E, F**) *C. briggsae *adults in a rotting apple (O635) from Orsay, showing bacterial proliferation in the intestinal lumen, with total obstruction in (F). (**G**) *C. elegans *adult in a rotting *Arum *stem from Plougasnou, with defects in feeding, accumulating bacteria in the pharyngeal lumen. The posterior pharyngeal bulb is visible in (E-G). The ventral side of the animals is down in all pictures.

Concerning nutrition, *C. elegans *is cultured in the laboratory on the bacterium *E. coli *OP50 and a supplement of cholesterol that the bacteria do not provide. Observations of *C. elegans *and *C. briggsae *animals freshly isolated from natural sources revealed that their intestinal lumen often contained eukaryotic cells, mostly yeasts (Figure 3A-D). The yeast cells sometimes appeared digested, with only cell wall remains, suggesting that *C. elegans *and *C. briggsae *may feed off them as well as off bacteria.

Wild *Caenorhabditis *were observed to often harbor a live bacterial flora in their intestinal lumen, as shown in Figure 3. In some instances, the flora proliferates and a large plug of bacteria obstructs the whole intestinal tract (Figure 3F). In some freshly isolated wild animals, pumping has in addition been observed to be hindered by bacteria that obstruct the anterior part of the alimentary tract, in front of the posterior bulb grinder (Figure 3G). The distinction between bacteria being food or pathogens is difficult to make in such cases.

**Figure 4:**
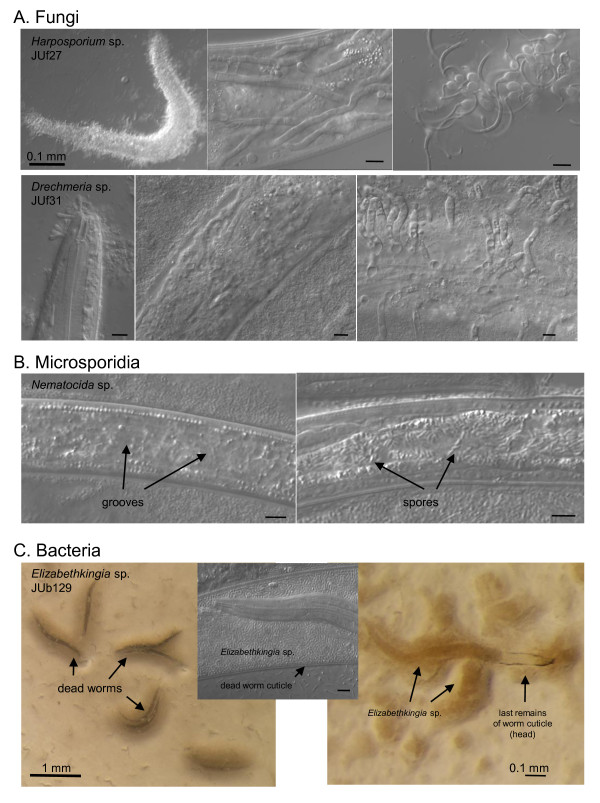
**Pathogens in natural *C. elegans *and *C. briggsae *populations**. (**A**) Fungal pathogens. Top three panels: The nematophagous fungus *Harposporium *sp. JUf27 was isolated from this dead *C. elegans *individual O143.12 (29 October 2008). JUf27 can infect the intestine of *C. elegans *N2 and produce hyphae that invade the whole body, resulting in death within six to eight days (middle panel). New spores are then produced at the surface of the dead nematode (right panel). The host response that it provokes has been characterized transcriptionally [23]. Bottom three panels: Fungal pathogen JUf31 in Orsay apple O641 on *C. briggsae*, *Drechmeria coniosporia*. Another *D*. *coniosporia *strain JUf28 was isolated from apple O567 on a *Pristionchus *sp. and deposited as CBS129433 at the CBS Fungal Biodiversity Centre, Utrecht, The Netherlands by Nathalie Pujol. These spores also develop into hyphae that invade the nematode body (middle panel) and produce a new generation of spores at the surface of the dead nematode (right panel). (**B**) Microsporidial infection in *C. elegans *in Orsay apple O695. Two infected dauer juveniles, in the microsporidial groove stage (left) and in the spore stage (right) (see [21] for microsporidial stages). Both groove and spore stages were also seen in *C. elegans *L2 larvae in apple O575. (**C**) Bacterial pathogen *Elizabethkingia *sp. (strain JUb129), found on *C. elegans *in Orsay apple O675, here shown on *C. elegans *N2. The bacteria induce worm bagging and then dissolve their cuticle. On the right, a larva is seen within her mother's corpse. Bars: 10 μm, except otherwise indicated.

We found several other types of pathogens in these natural populations. The first *C. elegans *natural virus was found during this survey in animals from Orsay apple O87 [20]. We previously reported microsporidia in *C. elegans *from an apple near Santeuil and in Montsoreau [21]. Consistent with our previous observations that microsporidia are relatively common natural parasites of *C. elegans *[20], microsporidia were observed in the surveyed *C. elegans *populations in Orsay apples in December 2009, October 2010 and November 2011 (one in six, 22 and ten *Caenorhabditis *populations, respectively [see Additional File 1; Figure 4B]), and in a stem in Plougasnou in August 2009 (one in seven populations [see Additional File 2]). As predators, trapping fungi with adhering knobs or rings are commonly found (for example, Figure 2 in [1], showing fungus JUf26). In addition, we observed fungi that develop hyphae from spores that are either ingested by the nematode or adhere to its cuticle, such as *Harposporium *sp. or *Drechmeria coniospora *[22,23] (Figure 4A). Bacteria that appear pathogenic from their effect on *Caenorhabditis *morphology, behavior and rate of proliferation are frequent and most remain to be characterized. In the most dramatic infection (Figure 4C), the bacterium first induces worm bagging (hatching of the embryos inside their mother) and then dissolves the worm cuticle, killing the entire population on the plate within about four days. The bacterium (strain JUb129) was isolated by culturing on *C. elegans *N2 and identified by 16S sequencing to be an *Elizabethkingia *sp. (Bacteroidetes; Flavobacteriaceae, formerly part of *Chryseobacterium *[24]). Bacteria of this genus are known to be able to digest keratins [25] and the present strain seems to be able to digest the collagens of the nematode cuticle.

### Spatio-temporal differences in distribution: studies in the Orsay orchard

In order to assess the relative spatio-temporal distribution of *C. briggsae *and *C. elegans *in a given location, we undertook a systematic sampling of rotten apples in the Orsay orchard. We sampled 20 to 28 apples throughout the orchard at 19 time points over four seasons (2008 to 2011) and, within a few hours of sampling, analyzed the distribution of developmental stages of *Caenorhabditis *in each of them. In addition, we sampled adjacent groups of apples twice in October 2008 (see analysis below). From positive samples, we isolated 12 to 25 (when available) individual *Caenorhabditis *and determined the species to which each belonged. During this survey, we also found a new male-female *Caenorhabditis *sp., *C*. sp. 13 [11].

**Figure 5:**
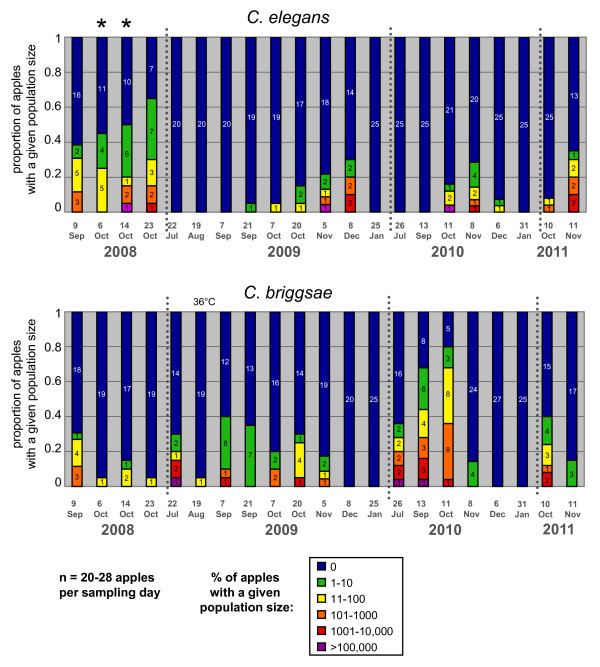
**Temporal distribution of *C. elegans *(top) and *C. briggsae *(bottom) abundance in Orsay apples along four consecutive years**. The graphs show the proportion of apples with a given census size (expressed on a log scale) of *C. elegans *or *C. briggsae *populations. The number of apples with a given census size is indicated in each color-coded bar. The two time points labeled by stars (6 and 14 October 2008) were sampled under a single tree (see Additional File 4) and were not used with the others in the statistical analyses. 19 August 2008 was particularly hot (36°C maximum temperature, labeled above the graph). See statistical analyses in the Results.

Both *C. briggsae *and *C. elegans *were abundantly found every year in the orchard. The two species could be found in the same apple (11 out of 429 apples). However, considering all individual apples, no positive correlation between the *C. elegans *and *C. briggsae *abundance in a given apple could be found (Spearman rank correlation, r = -0.057, *P *= 0.23). The temporal distribution of *C. briggsae *and *C. elegans *is presented in Figure 5, and their spatial distribution in Additional File 3.

For each species, both day of sampling and individual apple were significant explanatory factors (generalized linear model, for *C. elegans*, main effect *day *treated as factor: df = 18, χ^2 ^= 192.19, *P *< 0.0001, *apple *nested in *day*: df = 19, χ^2 ^= 44.63, *P *= 0.0008; for *C. briggsae*, main effect *day*: χ^2 ^= 255.22, *P *< 0.0001, *apple *nested in *day*: χ^2 ^= 48.09, *P *= 0.0003). Each species showed a significant temporal distribution along the season, an effect of the year and a significant interaction term (Generalized Linear Model, for *C. elegans*, main effects *year*: df = 3, χ^2 ^= 67.36, *P *< 0.0001, *month*: df = 1, χ^2 ^= 17.387, *P *< 0.0001, year*month: df = 3, χ^2 ^= 20.14, *P *= 0.0002; for *C. briggsae*, main effects *year*: χ^2 ^= 11.75, *P *< 0.0001, *month*: χ^2 ^= 127.69, *P *< 0.0001, year*month: χ^2 ^= 34.16, *P *< 0.0001). Indeed, the temporal distribution of the two species did not coincide (Spearman's rank correlation of cumulative abundance indices at one time point, r = -0.075, *P *= 0.76). *C. briggsae *was found alone in summer and *C. elegans *only appeared in September to early October. *C. briggsae *faded out from October on. *C. elegans *was then predominant in November and finally faded out in December to January (Figure 5). The third species, *C*. sp. 13, was only found once in September 2008, twice (on the Eastern orchard end) in November/December 2010 and once in November 2011 [see Additional Files 1 and 3].

At a time when both species were present, we screened for *C. elegans *and *C. briggsae *at a smaller spatial scale, by sampling two sets of apples, each below one tree, in four groups of five apples. The distance between apples within one group was 0 to 20 cm, and the distance between groups was 0.6 to 2.5 m [see Additional File 4]. Each apple was cut in the field into four pieces (when its shape and rotting stage allowed it), namely top, bottom, lateral and inner sides, and each part was separately analyzed in the laboratory [see Additional File 1]. There was no effect of day on the abundance index of each species in this dataset, and a strong effect of group, at least for *C. elegans *for which the sample size was larger (generalized linear model, for *C. elegans*, main effect *day*: df = 1, χ^2 ^= 0.761, *P *= 0.38, *group *nested in *day*: df = 6, χ^2 ^= 32.11, *P *< 0.0001; for *C. briggsae*, main effect *day*: χ^2 ^= 1.33, *P *= 0.25, *group *nested in *day*: χ^2 ^= 12.68, *P *= 0.048). Thus, at the scale of the apple groups, the distribution of *Caenorhabditis *individuals was heterogeneous, indicating that migration among groups (at the scale of 1 m) was limiting. Molecular analysis of the isolated worms will eventually provide more power to detect spatial patterns. Within apples that contained *C. elegans*, individuals were preferentially found at the bottom of the apple (towards the soil) compared to the top (Wilcoxon signed rank test, *P *= 0.0054, n = 15). Such a distribution was not found for *C. briggsae*, not even a similar trend; however, sample size was low (*P *= 0.875, n = 4 apples with *C. briggsae *in this set).

### Temperature affects the relative abundance of *C. elegans *versus *C. briggsae *in laboratory competition experiments

**Figure 6:**
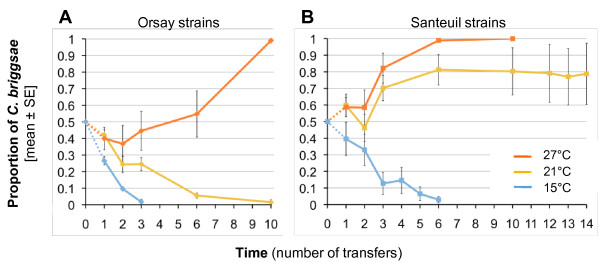
**Experimental evolution of the proportion of *C. briggsae *individuals in competition with *C. elegans *at different growth temperatures**. Two wild strains of *C. elegans *and *C. briggsae *from Orsay (**A**) or Santeuil (**B**) were competed against each other in the laboratory for several generations at three different temperatures (15°C, 21°C and 27°C). Starting from a frequency of 50% (ten *C. elegans *L4 larvae and ten *C. briggsae *L4 larvae), the proportion of *C. briggsae *individuals was quantified at different time points with five replicate populations per treatment. The mean proportion across replicates is indictaed as a thick line, and error bars indicate standard errors over replicates. Replicates are indicated as thin dotted lines. Time is represented as the number of transfers of each population to a fresh culture plate from the beginning of the experiment. For each treatment, the experiment was continued until all replicates reached fixation of either *C. briggsae *or *C. elegans*. With the Santeuil strains at 21°C, *C. briggsae *outcompeted *C. elegans *in four out of five replicates.

Several ecological parameters could account for the observed different seasonal distribution of *C. elegans *and *C. briggsae *in the Orsay orchard. Temperature is an obvious candidate as it varies between summer and winter in France (from an average of 19.2°C in August to 3.3°C in December over 2008 to 2011 in the Orsay area) and the range of temperature allowing fertility in the laboratory is lower in *C. elegans *compared to *C. briggsae *[26-28]. In order to show that temperature can affect the distribution of the two species in a given habitat, we performed competition assays between *C. elegans *and *C. briggsae *populations grown at 15°C, 21°C and 27°C in a controlled laboratory environment. Two different wild isolates of *C. elegans *and *C. briggsae *(either from Orsay or Santeuil) were competed in parallel at each temperature. These two pairs of sympatric strains were chosen arbitrarily among the wild isolates of *C. elegans *and *C. briggsae *to test for the effect of species, but not to study sampling site effects (to this end, more pairs of strains should have been compared). Overall, six different combinations of temperature and genotype were assessed and for each one, five replicate cultures were grown in parallel and transferred to fresh plates at fixed intervals of time to avoid starvation. The number of transfers was thus used as a measure of time in the analysis. Starting from an equal number of *C. elegans *and *C. briggsae *larvae, the proportion of both species was followed through time using quantitative pyrosequencing. As expected, for both pairs of genotypes (Orsay and Santeuil), the temporal dynamics of *C. briggsae *frequency depended on temperature (Figure 6). The competitive ability of *C. briggsae *versus *C. elegans *increased as the temperature increased from 15°C to 21°C (generalized linear model, main effect *temperature*: df = 1, χ^2 ^= 38.32, *P *< 0.0001, *time *nested in *temperature*: df = 1, χ^2 ^= 1.08, *P *= 0.298) and from 21°C to 27°C (generalized linear model, main effect *temperature*: df = 1, χ^2 ^= 8.15, *P *= 0.004, *time *nested in *temperature*: df = 1, χ^2 ^= 17.08, *P *< 0.0001). In addition, although the genotype of the wild isolates did not affect the direction of the temperature effect, it significantly changed the dynamics of species frequency (Figure 6). Indeed, for each temperature condition, the competitive ability of *C. briggsae *versus *C. elegans *is always higher for the Santeuil strains compared to the Orsay strains, resulting in a significant genotype effect and in a non-significant genotype × temperature interaction (generalized linear model, main effect *genotype*: df = 1, χ^2 ^= 4.86, *P *< 0.0001, genotype*temperature: df = 1, χ^2 ^= 4.41, *P *= 0.086). Strikingly, the *C. elegans *strain from Orsay was fixed in all replicate plates after ten transfers (360 hours) when competed against *C. briggsae *from Orsay at 21°C (Figure 6A), whereas at the same temperature, *C. elegans *from Santeuil disappeared in four out of five replicate plates when competed against *C. briggsae *from Santeuil (Figure 6B).

Compared with *C. briggsae*, the *C. elegans *strains used in the competition assays reached sexual maturity at a lower age at 15°C, but at a higher age at 21°C and 27°C [see Additional File 5]. Therefore, phenotypic variation in minimal generation time between the two species might contribute to the effect of temperature on the temporal dynamics of mixed populations of *C. elegans *and *C. briggsae*. In contrast, the effect of the genetic background is not likely to be explained by differences in the age at maturity, as this trait does not vary between the two *C. elegans *strains or between the two *C. briggsae *strains for all temperatures [see Additional File 5].

These results confirm that temperature is a relevant parameter to explain the seasonal distribution of *C. elegans *and *C. briggsae *and suggest that its fitness effect could partially be mediated through variation in minimal generation time. However, temperature is not sufficient to predict the relative abundance of the two species, since for intermediate temperatures the species found in the majority was shown to depend on the genetic background of the strains.

## Discussion

### Natural habitat of *C. elegans *and *C. briggsae*

Here we provide the first detailed analysis of natural populations of the nematodes *C. elegans *and *C. briggsae *in a habitat where they feed and proliferate. We identified rotting fruits and stems of herbaceous plants as natural habitats where *C. elegans *can be found feeding and proliferating in large numbers. In contrast to tropical areas where large wild fruits are easily sampled [11], the main source of large fruits in mainland France is provided by domesticated plant species. In temperate areas, rotting stems of herbaceous plants may be a more natural habitat of *C. elegans*.

### Individual and population stage

Scoring a large number of natural populations of *C. elegans *and *C. briggsae *in rotting fruits and stems provided a comprehensive overview of individual (that is, developmental stage) and population stages (as assessed by the presence and relative frequency of developmental stages). Populations in compost were previously found to be mostly composed of dauer larvae. Here in rotting fruits and stems, we found for the first time populations without any dauer larvae, usually with an intermediate census size of 10 to 1,000 individuals [See Additional Files 1 and 2]. Larger populations of 1,000 to 100,000 individuals included pre-dauers (L2d) and dauers. The stages of individuals in small populations could not always be assessed (because the individuals were not always seen within a few hours of sampling), yet some were seen to be composed of one or a few dauer larvae.

The analysis of groups of adjacent apples below a given tree showed heterogeneity at this scale in the distribution of *C. elegans *and/or *C. briggsae*. Thus, migration among groups (at a 1 m scale) was likely limiting. The fact that nearby apples may contain widely different population census and stages [See Additional File 1, 6 October and 14 October 2008] suggests that proliferating populations mostly remain in the same fruit.

From these population snapshots, one can infer the likely dynamics of these populations. Migrating dauer larvae initiate a new population in a given fruit or stem, either being discharged from a large invertebrate or migrating at small range through the leaf litter or soil. Depending on habitat and available food, the dauer larvae may start a new population by staying in place, exiting the dauer stage and reproducing. Exit from the dauer stage is a key decision. Upon food exhaustion and crowding, young L1 larvae in the population may enter the pre-dauer and dauer stage. Entry in the dauer stage seems to occur quite synchronously for all young larvae in a population [See Additional Files 1 and 2]. It is so far unclear how many generations are supported by a given fruit or stem, but from the larger observed populations, at least two to three generations must be required before all larvae are in the dauer stage in good conditions. Dauers may then leave the substrate, and only a few will make it to a good food source. *C. elegans *and *C. briggsae *thus typically have booming population dynamics, which selects for a short generation time and fast progeny production.

### Associated micro-organisms and resource for further studies

The organisms occurring in the habitats where *C. elegans *and *C. briggsae *feeding stages are found are likely to be important players in their ecology, either as potential food or as pathogens. Pathogens may enter through the digestive tract or else adhere to the nematode cuticle.

We here show that wild-caught *Caenorhabditis *often harbor a large microbial fauna in their intestinal lumen. The N2 reference strain of *C. elegans *cultured on *E. coli *OP50 in standard laboratory conditions crushes bacteria in its pharyngeal grinder almost fully efficiently. In older laboratory adults (starting at two days of adulthood), some live *E. coli *may eventually succeed in entering the intestinal tract, escaping defecation and starting to colonize and proliferate in the intestinal lumen [29,30]. *E. coli *can enter the nematode intestine much earlier in *C. elegans phm-2 *mutants with a defective grinder [29]. Some (human) pathogenic bacteria can pass the grinder of *C. elegans *N2 and colonize its intestinal lumen [29,31-33], perhaps by regulating the production of enzymes that affect it [33,34]. The absence of live bacterial cells in the intestinal lumen is thus considered the norm for laboratory *C. elegans*. In contrast, wild animals were observed to harbor a live bacterial flora in their intestinal lumen. The flow of bacteria is regulated by four dynamic components, namely, i) entry past the pharyngeal valve and grinder, ii) proliferation, iii) disappearance by digestion or host defences, and iv) exit through the rectum with worm defecation. In many cases, a healthy steady-state of bacterial flow appears to result from the balance between these four components. In some instances however, proliferation takes over and the obstructed intestinal tract can no longer support any flow (Figure 3F). Such weak and strong colonization phenotypes can sometimes be maintained over several generations and depend both on the microbial fauna and on the worm genotype: many wild *C. elegans *isolates were not as efficient in crushing *E. coli *OP50 as *C. elegans *N2 is, and many wild bacteria could colonize *C. elegans *N2 more readily than *E. coli *OP50 does (MAF, unpublished observations). The dynamics of microbe entry, growth, digestion, and exit will be interesting to study, as well as to determine the metabolic and pathogenic effects of different intestinal microbial communities.

*C. elegans *N2 cultured in standard laboratory conditions activates its pharyngeal muscles in a rapid cycle, pumping several times per second in the presence of food [35]. Pumping of wild-caught animals could occasionally be observed to be hindered by bacteria obstructing the pharynx (Figure 3G). Such animals appeared malnourished, as indicated by the pale coloration of their intestine. The case in Figure 3G appears to result from an interaction between the bacteria in the samples and the *C. elegans *wild genotype, because neither *C. elegans *N2 fed on the corresponding wild bacteria nor the wild *C. elegans *genotype fed on *E. coli *OP50 displayed this defective swallowing phenotype.

Fungi are abundant in rotting fruits and stems, and could be seen in the intestines of wild-caught *Caenorhabditis*. Yeasts synthesize sterols such as ergosterol and dehydroergosterol (for example [36] for *Saccharomyces cerevisiae*), which can be used as a sterol source by *C. elegans *[37]. It is thus possible that *C. elegans *derives its sterols from fungi. Alternatively, it may ingest sterols from plant remains as well.

We here established a large collection of isolates of *C. elegans*, and to a lesser degree *C. briggsae*, which was generally frozen without bleaching, thus potentially retaining associated microbial fauna. We also directly froze a fraction of the original sample at -80°C for further analysis. This opens the way for evolutionary and ecological characterization, such as population genetic analysis of the nematodes, metagenomics and isolation of the microbial fauna, and surveys of the occurrence of pathogens.

### Habitat sharing: *C. elegans *likes it cool

*C. elegans *shares its habitat with *C. briggsae*, and also with a variety of other bacterivorous nematodes of various genera in the families Rhabditidae (including diplogastrids such as *Pristionchus*) and Panagrolaimidae (*Panagrellus*, *Panagrolaimus*). These species are likely to compete for food, and some of them, such as *Pristionchus *species [38], may also act as predators of *Caenorhabditis *species, although this remains to be demonstrated.

Most strinkingly, we find *C. elegans *and *C. briggsae *in the same type of habitat. However, several results suggest that *C. elegans *is preferentially found at cooler temperatures than *C. briggsae*. First and most strikingly, the seasonal pattern in the Orsay orchard is strong, with *C. briggsae *found in summer and early fall and *C. elegans *in the late fall. Second, the Plougasnou area in Brittany was sampled over the years in many places, and particularly in high summer when only *C. briggsae *could be found in Orsay. This Western France region presents an oceanic (wet, cool, quite even throughout the year) climate and only yielded *C. elegans*. Both species were found in all other tested regions of France, North, South and East.

Both species are found on several continents, but *C. briggsae *is found in both tropical and temperate regions whereas *C. elegans *is found mostly in temperate regions, or in tropical regions at relatively high altitudes [11]. The latter might be explained by decreased temperatures or by other correlates of altitude, such as oxygen levels. Deeper sampling will be required to confirm the tendency of *C. elegans *to live at high altitude in tropical locations.

The laboratory competition assays performed in controlled environments clearly show an effect of growth temperature on the relative fitness of *C. elegans *and *C. briggsae *when sharing the same habitat. Consistent with the seasonal and worldwide patterns, *C. briggsae *wins at higher temperatures and *C. elegans *at lower temperatures (Figure 6). In these competition experiments, the effect of temperature is likely mediated in part by the difference in generation time between the two species [See Additional File 5]. Other temperature-sensitive traits of *C. elegans *and *C. briggsae *can be inferred from comparisons of independent studies in each species and may also contribute to the competition results. For example, self-brood size declines in *C. elegans *at temperatures above 18 to 20°C [28,39], whereas the decline occurs at higher temperatures in *C. briggsae*, with still a high brood size at 28°C [27], a temperature where *C. elegans *is fully sterile. Dauer entry is another key developmental decision that is differentially regulated by temperature in *C. elegans *and *C. briggsae *and thus could be involved in the spatio-temporal dynamics of the two species in wild populations. A growth temperature above 27°C is sufficient to trigger dauer entry in *C. elegans *wild isolates with various penetrances, whereas it seems to have no effect in *C. briggsae *[40,41].

Our data further indicate an effect of the genotype within a species in the competition experiment. Other competition experiments will be required to determine which species is involved in this genotype effect, which may be due to a difference of fitness between the two *C. elegans *strains, the two *C. briggsae *strains, an independent combination of both, or a specific interaction. This intraspecific difference between the two sets of genotypes is unlikely to be explained by a difference in generation time [See Additional File 5]. Each species was shown to display intraspecific variation in the temperature that maximizes lifetime fecundity of wild isolates [27,28,39]. In *C. briggsae*, the temperature resistance correlates with the latitude of strain isolation [27]. In *C. elegans*, a few quantitative trait loci (QTLs) involved in the thermal plasticity of fertility, age at maturity, growth rate and egg size have been mapped from a set of recombinant inbred lines (RILs) between N2 and the Hawaiian strain CB4856 [42]. The plasticity of gene expression to temperature was shown to vary between N2 and CB4856, primarily due to *trans*-acting eQTLs [43]. Finally, a third analysis from the same RILs led to the identification of a coding polymorphism in the *tra-3 *gene that affects body size response to temperature [44]. In the wild, besides an effect of these life history traits, the temperature-dependent distribution of *C. elegans *and *C. briggsae *could also be regulated through variation in behavioral responses. Interestingly, when placed in a temperature gradient, different isolates of *C. elegans *present different thermotactic behavior and seem to show a preference for the temperature maximizing their fitness [28]. The reference strain N2 could have lost this adaptive thermophilic response after introduction to the stable temperature of the laboratory [45]. A better understanding of how thermotactic behaviors evolve in the wild will thus require a better characterization of the spatial and temporal dynamics of temperature in natural habitats of *C. elegans*.

Apart from temperature, the population dynamics of *Caenorhabditis *species is likely to be affected by other ecological parameters that are not yet identified. We note for instance that the distribution of *Caenorhabditis *species of the *Elegans *group in Europe is puzzling and does not quite fit a simple temperature pattern. Indeed, *C. briggsae *has only been found so far in France (not more Southern countries, such as Spain and Portugal, where *C. elegans *was found). *C. remanei *instead is found in Germany, Switzerland and Hungary [46] and the North-East of France (Alsace; Figure 1). Whether this pattern might be due to limited migration or results from aspects of local adaptation other than temperature sensitivity remains to be determined.

## Conclusions

*C. elegans *and *C. briggsae *were found to proliferate in the same rotting vegetal substrates. For the first time, we found proliferating populations without arrested dauer stages. The large populations, however, always contain pre-dauer or dauer stages. Together with the fact that these rotting substrates are temporary habitats, it appears thus likely that *C. elegans *and *C. briggsae *undergo successive population growth periods upon encountering a favorable rotting substrate, separated by periods of migration in the dauer stage. The discovery of habitats where *C. elegans *and *C. briggsae *feed opens the way for evolutionary and ecological characterization. Here we show that the temporal sharing of the habitat by *C. elegans *and *C. briggsae *coincides with their temperature preference in the laboratory, with *C. elegans *populations growing faster than *C. briggsae *at cooler temperatures, and *C. briggsae *faster at higher temperatures.

## Methods

### Nematode sampling and isolation

The samples were photographed and collected in plastic tubes (60 ml) or zipped bags. In the laboratory, they were weighed and cut into pieces (about 1 cm^3^). At least half of the sample was used for nematode isolation, while the rest was weighed and stored at -80°C for further use. Samples were analyzed on the day of sampling for all Orsay, Santeuil and Ivry time points. The day of sample deposition on plate and first analysis is indicated for Le Blanc and Plougasnou samples in Additional File 2.

The samples were placed onto standard *C. elegans *Normal Growth Medium culture plates [47] of 90 or 55 mm diameter, previously seeded with an *E. coli *OP50 lawn in the center. The samples were spread around the lawn and 1 to 2 ml moisture (water or M9 solution) was added onto the samples. All plates were examined regularly in the dissecting microscope: several times within a few hours, once or twice on the next two days, and at least on days 4 and 7. Sampled arthropods and mollusks were usually first left free on the plate for a few hours to test for the presence of nematodes on their surface, then sacrificed with a scalpel. Nematodes that crawled out of the sample were identified to the genus (or family) level by morphological criteria [47] in the dissecting microscope with trans-illumination. Animals resembling *Caenorhabditis *were picked individually onto 55 mm culture plates. Because i) 'sick' wild animals do not always resemble the canonical healthy laboratory animal and ii) dauer larvae are difficult to distinguish, animals of dubious genus were also isolated for further identification. *C. elegans *and *C. briggsae *are both facultative selfers and require a single hermaphrodite larva to reproduce (males are very rare). *Caenorhabditis *sp. 13, however, reproduces through males and females. In this case, cultures were started from an adult female or with a pair of male and female larvae.

### Species identification

*C. elegans *and *C. briggsae *resemble each other morphologically, even when observed with a high-power microscope. In the early part of this work, we systematically assessed the species using a cross and/or a molecular method.

For the biological species test, three to six hermaphrodites of the tested isolate were placed with three to six males of reference laboratory strains *C. elegans *N2 or *C. briggsae *AF16. The plates were observed three to four days later at 20° to 23°C to test for the presence of a large proportion (> 20%) of males in the progeny, an indication of a successful cross [48].

Molecular tests were as described in [47], using species-specific PCR primers. After testing hundreds of *Caenorhabditis *cultures, we noticed some morphological and behavioral criteria that were decisive for species identification, when assayed on populations, that is, not on the single wild parent, which remains hard to identify: 1) *C. elegans *retains more embryos in its uterus (usually 10 to 50) than *C. briggsae *(usually none to five); 2) *C. elegans *has a dumpier body aspect ratio than *C. briggsae*; 3) *C. elegans *clumps on culture plates with food, whereas *C. briggsae *does not. The latter criterion was rendered decisive when it was understood that the non-clumping phenotypes of the *C. elegans *reference strain N2 was acquired through laboratory mutations [49]. All three of the above criteria depend on the environment and are best scored in clean cultures, that is, with *E. coli *OP50 only. If the species identification was dubious, cultures were systematically either cleaned by bleaching or identified by crossing with males. All identifications that were dubious on morphological/behavioral criteria proved correct with genetic or molecular criteria. With training, most identifications could be made using morphological/behavioral criteria.

### Orsay orchard sampling

This orchard is located at 48.7015 North, 2.1725 East. This orchard contains 322 apple trees with a large diversity of apple varieties, which ripen and rot throughout the second half of the year (July to December), and a large fraction of the apples are left to rot (except in 2011 when they were harvested). Schematically, the main grid has trees spaced every 3 meters in each (lettered) North-South column and every 4 meters in each (numbered) West-East line, from A1 to S23, with an open space in the middle (222 trees in this grid). In addition, two columns of 2-meter-spaced trees, 2-meters apart, are found on the East side, after a 17 m wide empty area (100 trees).

At each of the 17 time points, 20 to 28 very rotten apples were sampled on the floor throughout the orchard, and the corresponding position noted. Apple production is very variable among trees and years, which may explain possible spatial patterns in sampling. Each apple was called "O" (for Orsay) followed by an identifying number. Similarly, Santeuil samples were labeled "S", followed by a number.

For each of the 6 and 13 October 2008 collections, a tree with a large number of rotten apples below it was chosen. Four groups (within 50 cm of each other on the ground) of five rotten apples were then chosen. When possible (that is, the apple was not yet reduced to a very flat puree), each apple was cut into bottom (towards soil at sampling time), top, lateral and inner pieces. Each piece was separately weighed and tested for nematode presence and census.

### *C. briggsae *versus *C. elegans *competition assays

Six competition assays involving two different pairs of *C. elegans *and *C. briggsae *wild strains cultivated at three different temperatures were performed in parallel for several generations to assess the role of temperature and genotype on the relative growth of the two species. For each of the six experimental conditions, five replicate populations were grown in parallel on 6 cm diameter NGM dishes (2.5% agar) seeded with 50 μl of OP50. We chose to compete the *C. elegans *strain JU1530 against the *C. briggsae *strain JU1529 (both isolated the same day from two different rotten apples found below the same tree in Orsay), and the *C. elegans *strain JU1918 against the *C. briggsae *strain JU2138 (isolated the same day from two different rotten stems in Santeuil). These strains were derived from wild parents by four to six generations of selfing in the laboratory, to avoid possible within-line heterozygosity due to outcrossing in the wild. The experimental populations were founded from ten L4 *C. elegans *individuals and ten L4 *C. briggsae *individuals deposited on each plate in the center of the *E. coli *lawn and grown at either 15°C, 21°C or 27°C. All strains were previously bleached and synchronized one generation prior to the experiment by 1.5 hour of egg laying of ten adults on eight plates.

To avoid starvation, a fraction of each population was transferred to a fresh culture plate at fixed intervals of time: worms grown at 15°C were transferred every 144 hours, while those maintained at 21°C or 27°C were transferred every 36 hours. Animals from old plates were retrieved in 1 ml of M9 solution in sterile conditions and centrifuged at 3,000 rpm for five minutes. The pellet (1 μl containing approximately 100 to 400 mixed stage individuals) was then dropped in the middle of a fresh culture plate.

For each replicate population, the proportion of *C. briggsae *versus *C. elegans *was quantified at different time points by pyrosequencing, using a PyroMark Q96 ID instrument from Biotage (Uppsala, Sweden). One single-nucleotide divergence was identified between the two species in an otherwise well-conserved region of the *eef-2 *coding sequence (C/T at position I:9,166,508 in WormBase WS228 release), allowing for non-biased PCR. For DNA preparation, 1.5 μl of M9 suspensions containing about 500 mixed-stage individuals from each culture plate were mixed with 18.5 μl of worm lysis buffer and proteinase K (100 μg/ml). After 1.5 hours of lysis at 60°C and 15 minutes of inactivation at 95°C, 0.5 μl of worm lysate were used as PCR template. PCRs were performed in 50 μl reactions containing 0.25 μl of GoTaq DNA polymerase (Promega, Madison, Wisconsin, USA), 10 μl of 5x GoTaq buffer, 5 μl dNTPs at 2 mM, 1 μl of forward primer at 10 μM (5'-GCTTACTTGCCTGTCAACGAGTC-3'), 0.17 μl of reverse primer at 10 μM (5'-TAGCAGGATACGACTATCCGGTGTTGGAGCGGAGAT-3') and 0.83 μl of a universal biotinylated primer at 10 μM (5'-[Btn]TAGCAGGATACGACTATC-3') [50]. The purification of single-stranded PCR amplicons and the pyrosequencing reactions were performed according to the manufacturer's instructions using the following sequencing primer: 5'-CTTCGGATTCACCGC-3'. *C. briggsae *versus *C. elegans *allele frequencies were obtained from the height of the pyrogram peaks using the Allele Quantification tool supplied with the pyrosequencing software of Biotage. For each of the six experimental conditions, the assay was stopped when all replicate populations were sufficiently close to fixation of either *C. elegans *or *C. briggsae *(more than 95% of the more frequent *eef-2 *allele).

To estimate the precision of the quantification method, the frequency of *eef-2 *alleles was measured on worm lysates prepared from known proportions of *C. briggsae *and *C. elegans *individuals. The discrepancy between observed and expected frequencies was, on average, 3.66%.

### Minimal generation time

The minimal generation time (or age from egg-laying to sexual maturity) was measured at 15°C, 21°C and 27°C for each *C. elegans *or *C. briggsae *strain used in the competition assays. A first pilot experiment was carried out to get a broad estimation of the age at which hermaphrodites became fertile at the different temperatures. Then, more accurate values of the age at maturity were measured as follows. The strains were synchronized by one to two hours of egg laying of ten adults at 15°C, 21°C and 27°C. For each strain, five larvae were isolated on separate 55 mm culture plates 24 hours (21°C and 27°C) or 48 hours (15°C) after egg laying. From 36 hours (27°C), 58 hours (21°C) or 92 hours (15°C) post-synchronization, plates were checked regularly for laid eggs until all isolated hermaphrodites reached reproductive maturity. The age at maturity was calculated as the difference between the time when the first eggs were observed and the time when the parent itself was laid (estimated as the middle time point of the one to two hour egg laying period).

### Data analysis

From the census observations, we assigned an *abundance index *for each species in each apple on a Log scale with an index of 1 for one to 10 individuals, 2 for 11 to 100, 3 for 10^2 ^to 10^3^, 4 for 10^3 ^to 10^4^, and 5 for > 10^4^. At each time point, a *cumulative abundance index *for each species was the sum of the individual index of the corresponding apples. Statistical analysis was performed in R version 2.11.1 [51].

To test whether the species abundance index varied along and across years, we used a generalized linear model (GLM), assuming a Poisson response variable and a log link function. Effects included in the model were *month *treated as a number and *year *treated as a factor. Year corresponded to a rotting apple production season, that is, from July to January. Month was thus calculated starting in July, allowing for half-integer numbers in cases of two time points in a month. Tested dependent variables included the *C. elegans *cumulative abundance index and the *C. briggsae *cumulative abundance index.

To test whether the distribution of apples was homogeneous among different groups of apples sampled below a given tree, we used a generalized linear model (GLM), assuming a Poisson response variable and a log link function. Effects included in the model were *day *treated as a factor and *group *nested in day, treated as a factor. Tested dependent variables included the *C. elegans *cumulative abundance index and the *C. briggsae *cumulative abundance index.

To test whether the abundance of the two species significantly differed, we used a Wilcoxon signed rank test on the weighted census of each species, corrected for the weights of each apple part. A Friedman rank sum test using the weighted census in all four apple parts (bottom, side, inside, top) was not significant for *C. elegans *(χ^2 ^= 6.6, df = 3, *P *= 0.0858).

To analyze the competition experiment, data were normalized using the arcsin function and a GLM was used, assuming a Gaussian response variable and an identity link function. Effects included in the model were *genotype *and *temperature *treated as factors, *time *(number of transfers) nested in *temperature*, and the interaction between *genotype *and *temperature*.

## Competing interests

The authors declare that they have no competing interests.

## Authors' contributions

MAF performed the sample collection and analysis. FD performed the competition experiment. MAF and FD wrote the manuscript. All authors read and approved the final manuscript.

## Supplementary Material

Additional file 1 **List of Orsay samples (orchard and woods), with census and stages of *C. elegans *(Ce, green), *C. briggsae *(Cb, red) and *C*. sp. 13 (blue) individuals**. Each sheet corresponds to a sampling date. The number of individuals of a given species is indicated in brackets in the column '*Caenorhabditis *species ID', expressed as a ratio over the total number of tested individuals. The samples are identified with the letter O for Orsay, followed by a number. Sample numbers are according to chronological order of sampling. D, dauer larva; ad, adult. For 6 and 14 October, the apple part is abbreviated with B = Bottom, I = Inside, S = Side, T = Top. Empty cells denote absence of *Caenorhabditis *species in the corresponding sample. Click here for file

Additional file 2 **List of samples from non-Orsay locations (indicated by sheet names: Santeuil, Ivry, Plougasnou, Le Blanc)**. Each sample identifier starts with a letter indicating the location (S, I, P, B, respectively). The table indicates the census and stages of *C. elegans *(Ce, green) and *C. briggsae *(Cb, red) individuals at different sampling dates. The number of individuals of a given species is indicated in brackets in the column '*Caenorhabditis *species ID', expressed as a ratio over the total number of tested individuals. In the Santeuil 31 Oct 2011 data, horizontal lines group samples that were isolated immediately next to each other, for example soil, moss and/or leaf litter next to a rotting stem (or two adjacent stems). Only one large proliferating population was isolated on that day, in a rotting stem (S168). Note that the Plougasnou area has been previously repeatedly sampled (mostly compost, isopods, snails) in [7,9] and also only yielded *C. elegans*. Click here for file

Additional file 3 **Spatial distribution of *Caenorhabditis *species in the Orsay orchard over 19 time points**. Black dots: apple trees. Apple positions were noted using the tree(s) they were closest to. Empty square: apple with no *Caenorhabditis*. The apples with *Caenorhabditis *are indicated with their sample name, for example "O11", and color-coded according to the species that was/were found. Red: *C. briggsae*. Blue: *C. elegans*. Mauve: *C. briggsae *and *C. elegans*. Yellow: *C*. sp. 13. Orange: *C. briggsae *and *C*. sp. 13. Green: *C. elegans *and *C*. sp. 13. The size of the square is proportional to the *Caenorhabditis *population size. Representative for each population size are 1 to 10 individuals: apple O14; 11 to 100: apple O13; 10^2 to ^10^3^: apple O15; 10^3 to ^10^4^: apple O145; > 10^4^: apple O535. The sampling date is indicated at the top left of each map; n: number of sampled apples [see Additional File 1 for scoring of these apples]. North is to the top. Click here for file

Additional file 4 **Spatial distribution of *Caenorhabditis *species at the scale of a few meters**. Twenty apples in four groups of five apples were sampled below a tree on (A) 6 October 2008 and (B) 14 October 2008. The tree trunk is labeled with a black circle. The relative position of each group is shown and the detailed position of apples within a group (within 50 cm of each other) is shown in a close-up in the adjacent rectangle. Blue: apple with *C. elegans*, with light blue for proliferating populations. Red: *C. briggsae*, with pink for proliferating populations. Large font size denotes large population size [see Additional File 1 for data]. Click here for file

Additional file 5 **Minimal generation time at 15°C, 21°C and 27°C of the four *Caenorhabditis *strains used in the competition assays**. The minimal generation time (or age at sexual maturity) was estimated from the observation of *N *= 5 hermaphrodites for each of the four wild isolates grown at the different temperature conditions. JU1530, JU1918, JU1529 and JU2138 are respectively *C. elegans *isolates from Orsay and Santeuil and *C. briggsae *isolates from Orsay and Santeuil. Error bars indicate the standard error (SE) of the mean over individuals. A two-way ANOVA (indicated below the graph) was performed to analyze the effect on the age at maturity of the main variables *species *and *temperature*, the effect of *strain *nested in *species *and the *species × temperature *interaction term. The box-Cox transformation was applied to the dataset to meet the assumptions of homogeneity of variances. We next performed post-hoc Tukey's HSD test on *strain *to determine groups of statistical significance. Two bars are significantly different (*P *< 0.05) if they are not labeled with a same letter. Within each species, the strain genotype has no significant effect on age at maturity at any temperature. However, the two *C. elegans *strains present a lower age at maturity than the two *C. briggsae *strains at 15°C, but a higher age at maturity at 21°C and at 27°C. As expected, for both species the minimal generation time decreases when temperature increases. Click here for file
