# Supplementary figures and images for: Population dynamics and habitat sharing of natural populations of Caenorhabditis elegans and C. briggsae
Source: BMC Biol. 2012 Jun 25;10:59. doi: 10.1186/1741-7007-10-59 (PMC3414772; doi:10.1186/1741-7007-10-59)

9 Sep 2008  
n=26

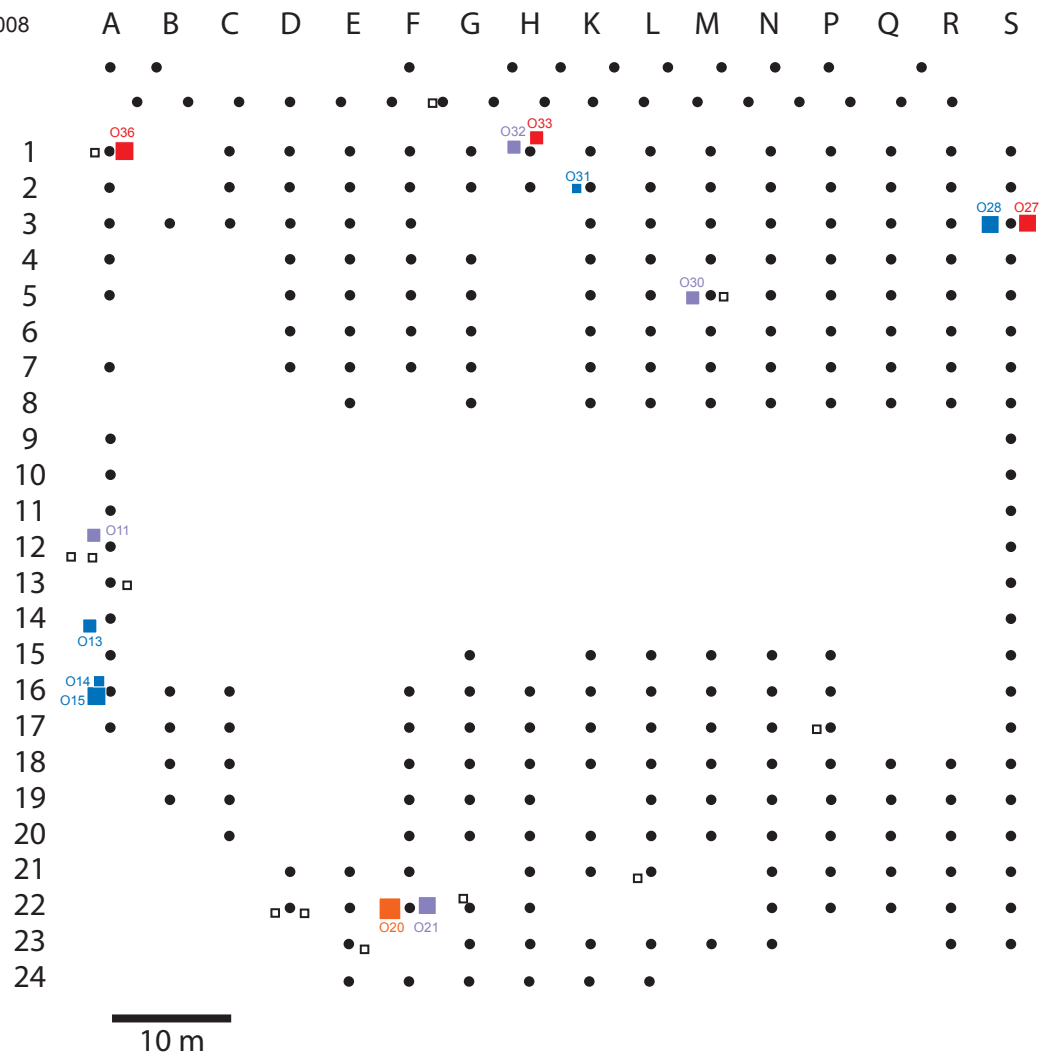

23 Oct 2008  
n=20

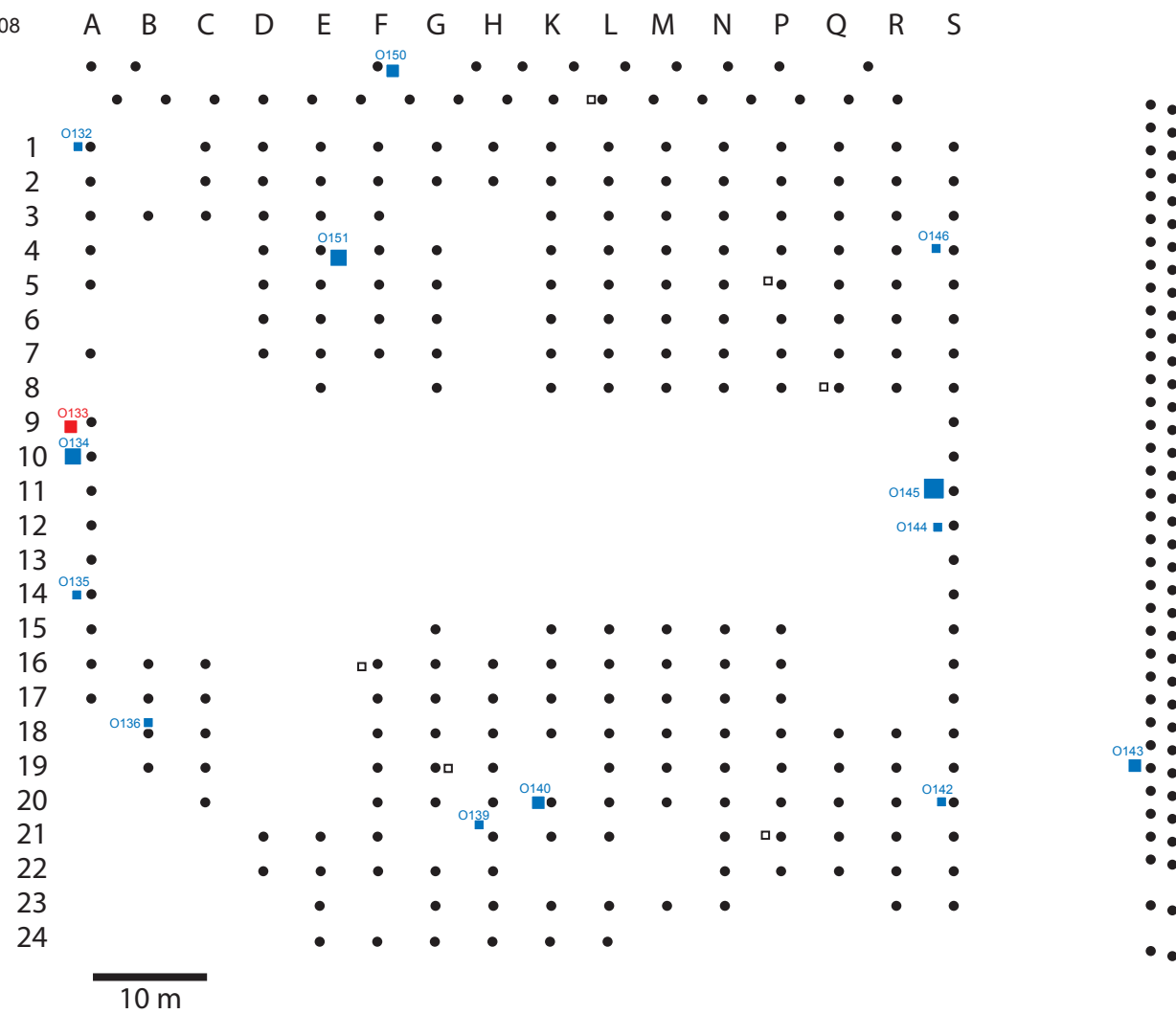



19 Aug 2009  
n=20

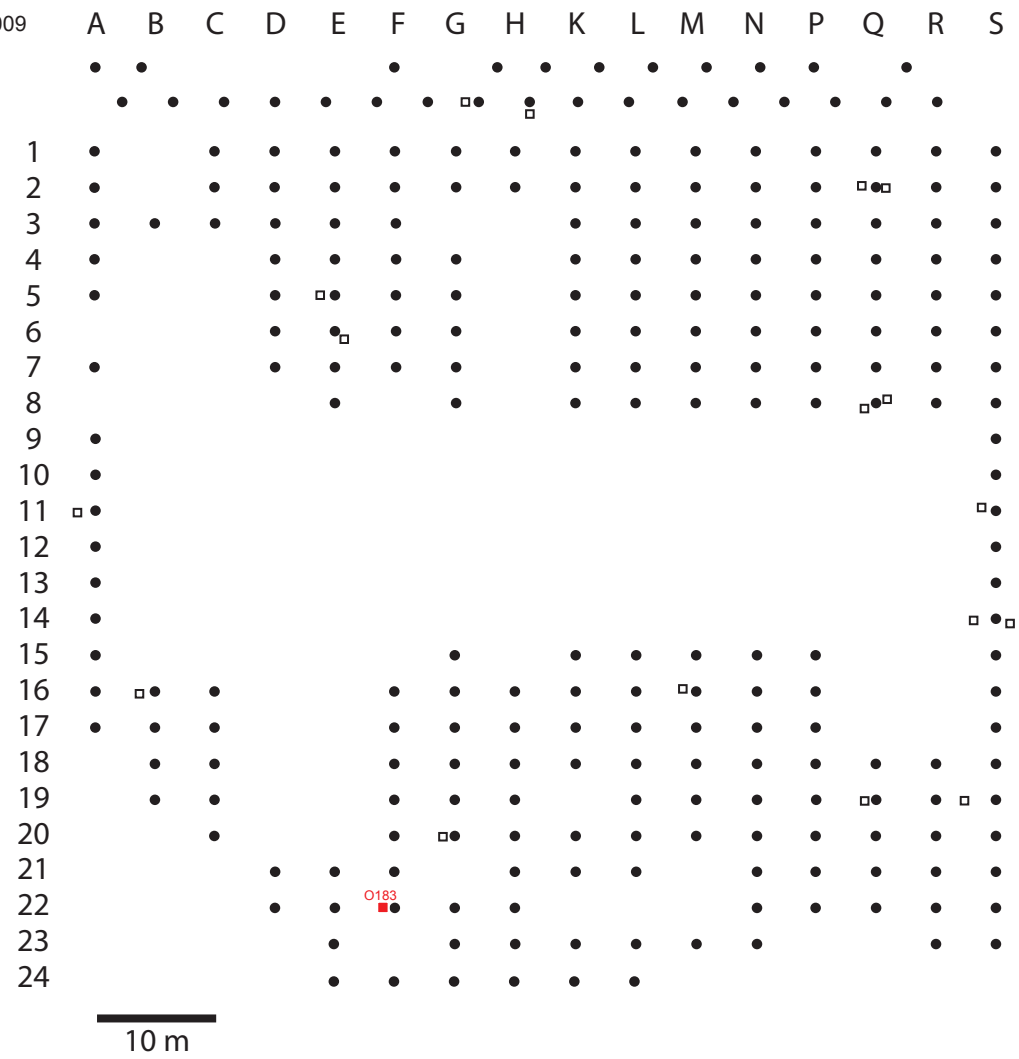

7 Sep 2009  
n=20

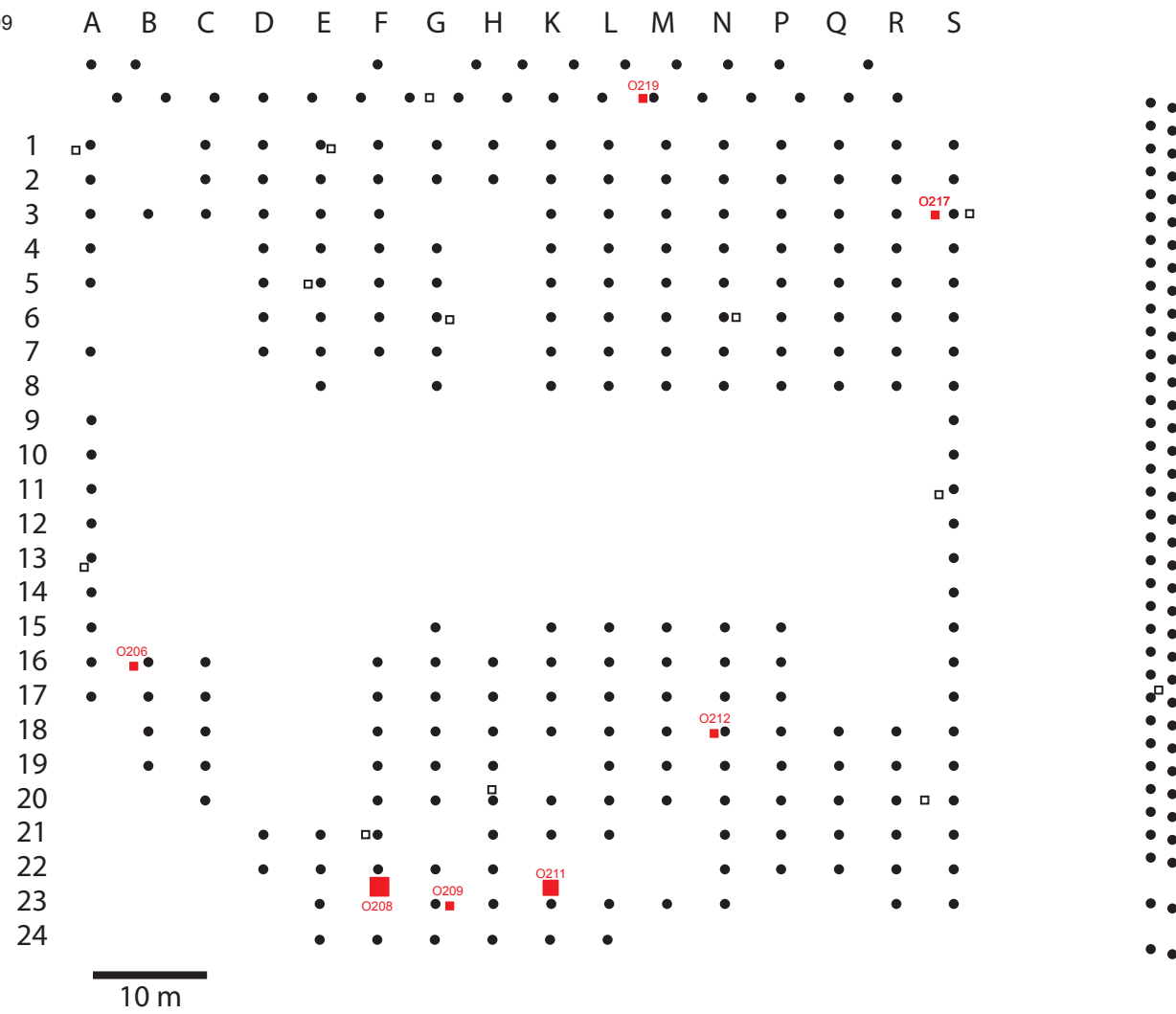

21 Sep 2009  
n=20

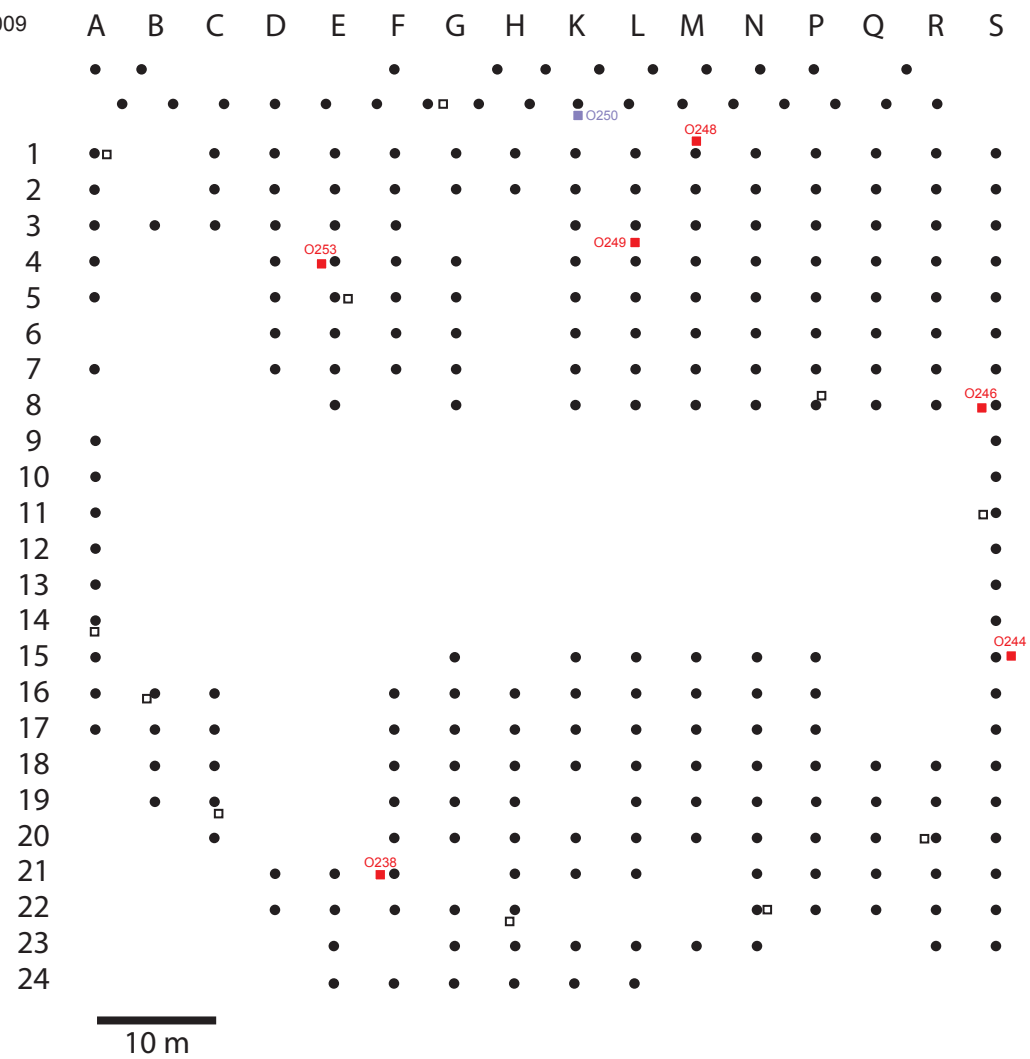

7 Oct 2009  
n=20

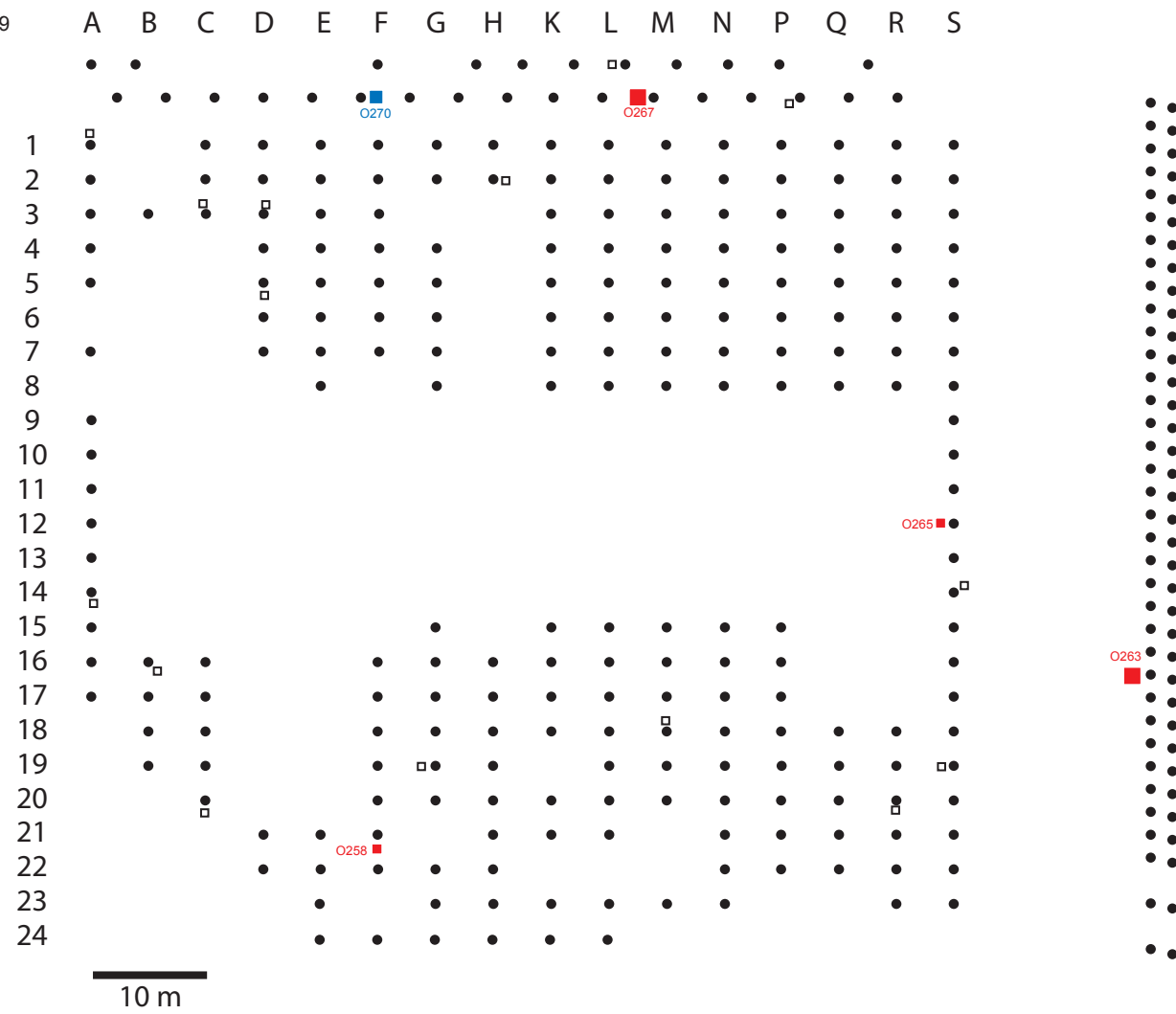

20 Oct 2009  
n=20

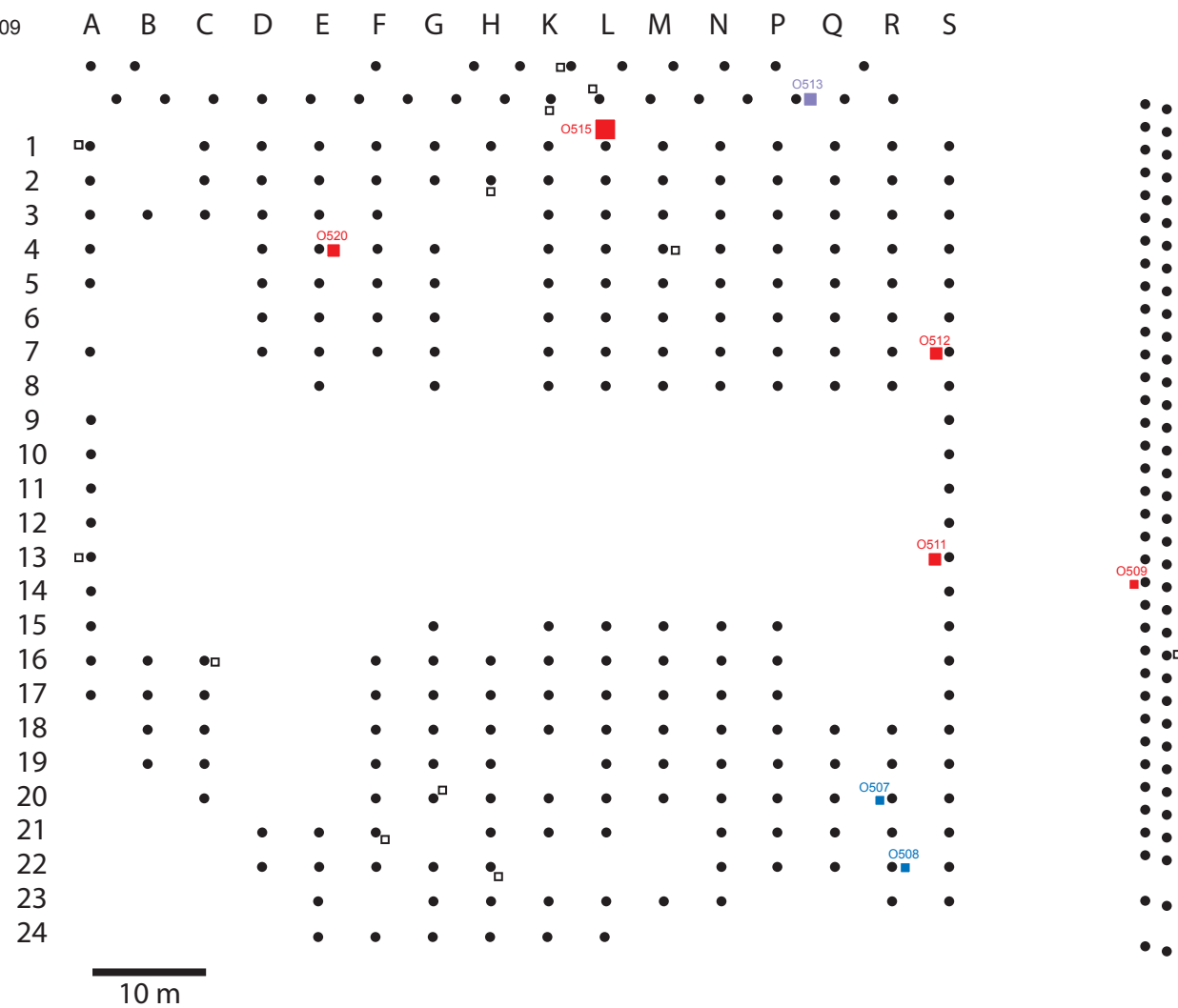

5 Nov 2009  
n=23

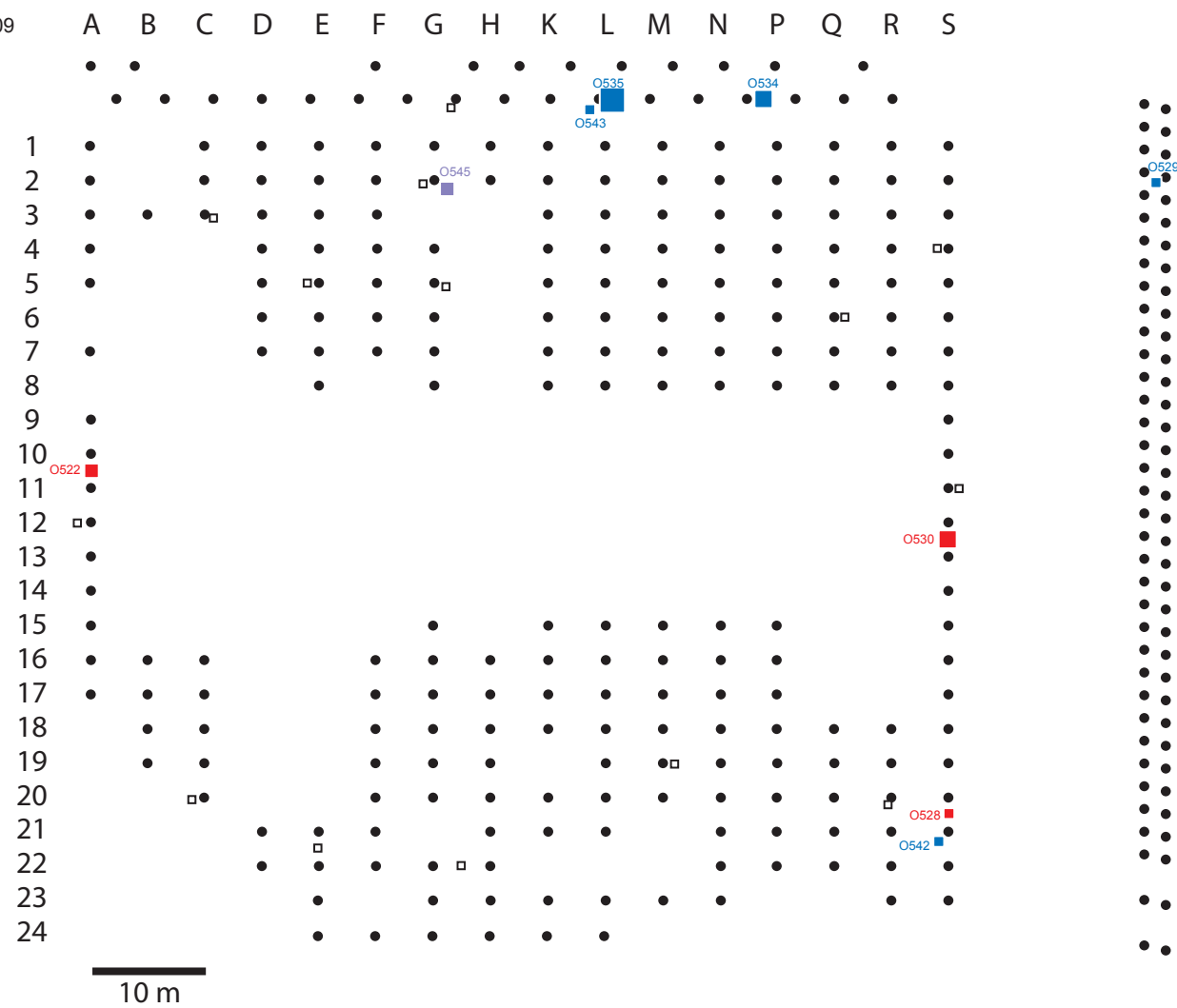

8 Dec 2009  
n=20

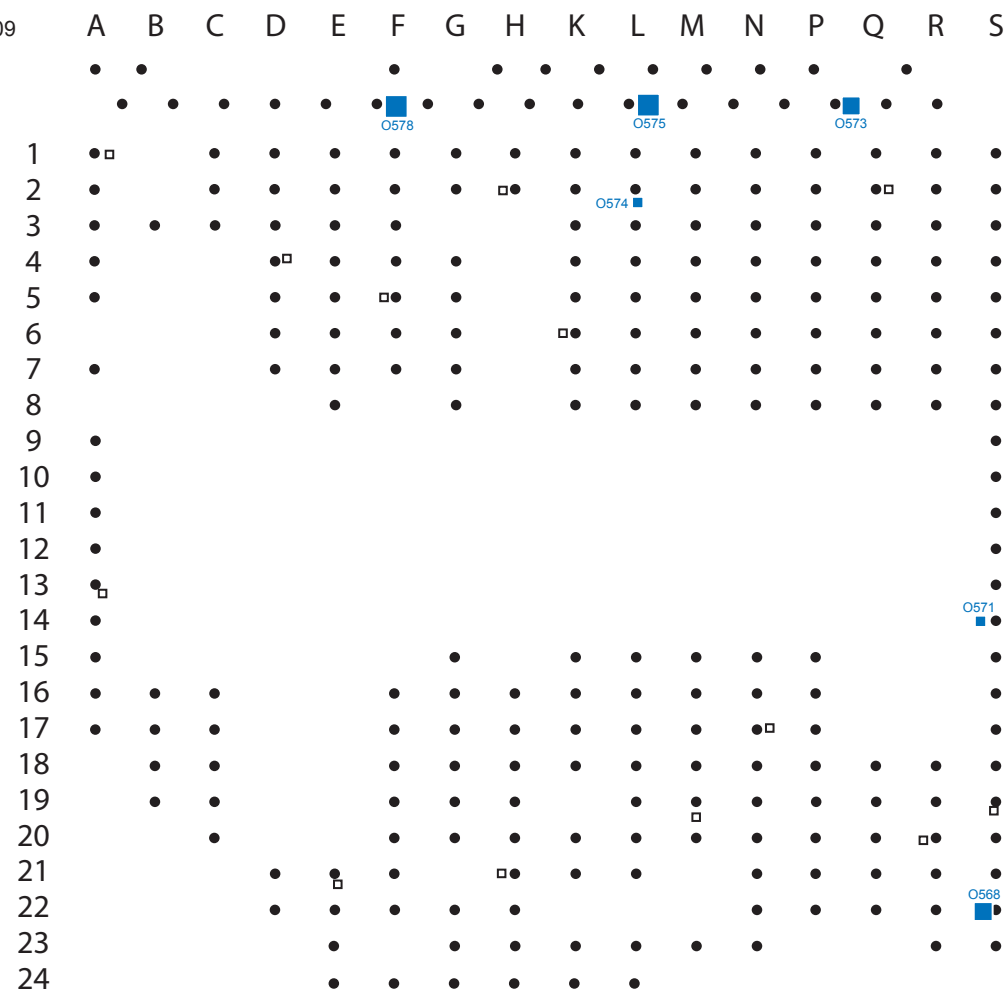

10 m

25 Jan 2010  
n=25

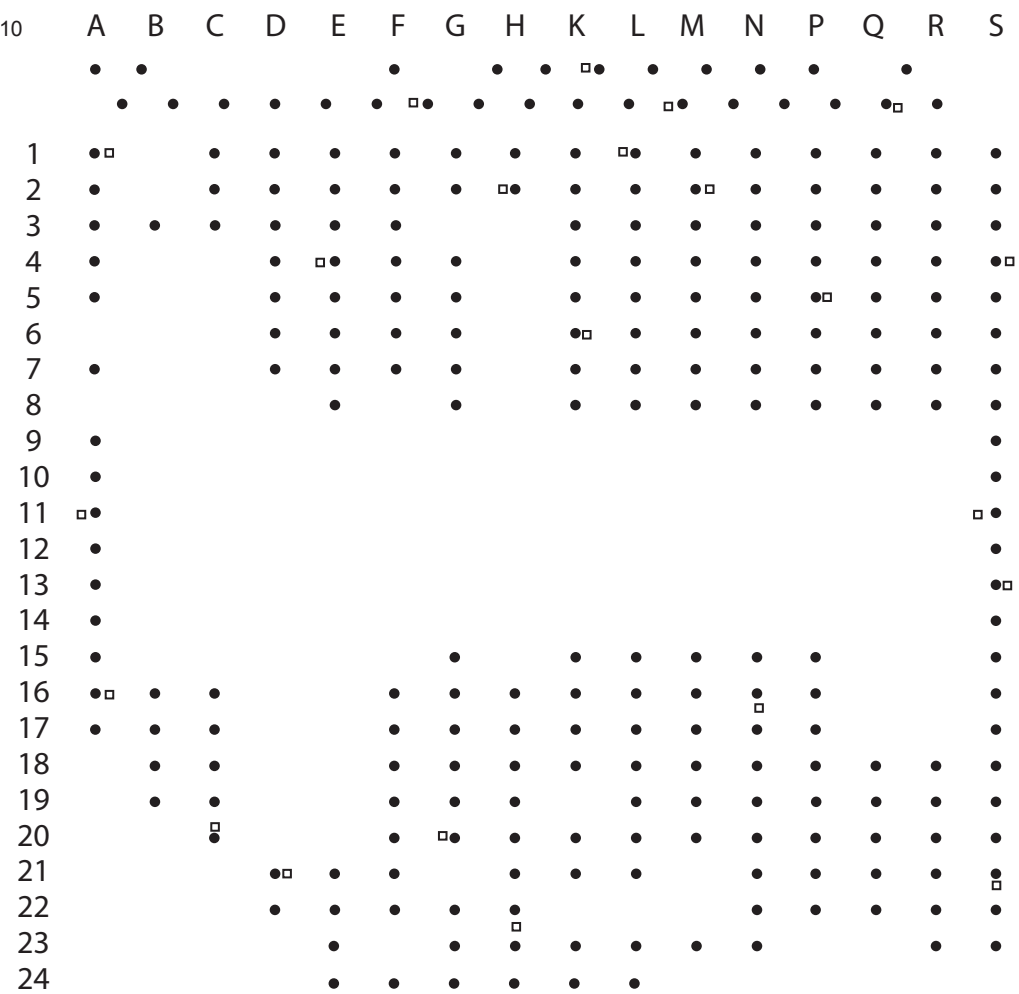

10 m

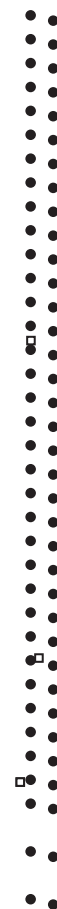

26 Jul 2010  
n=25

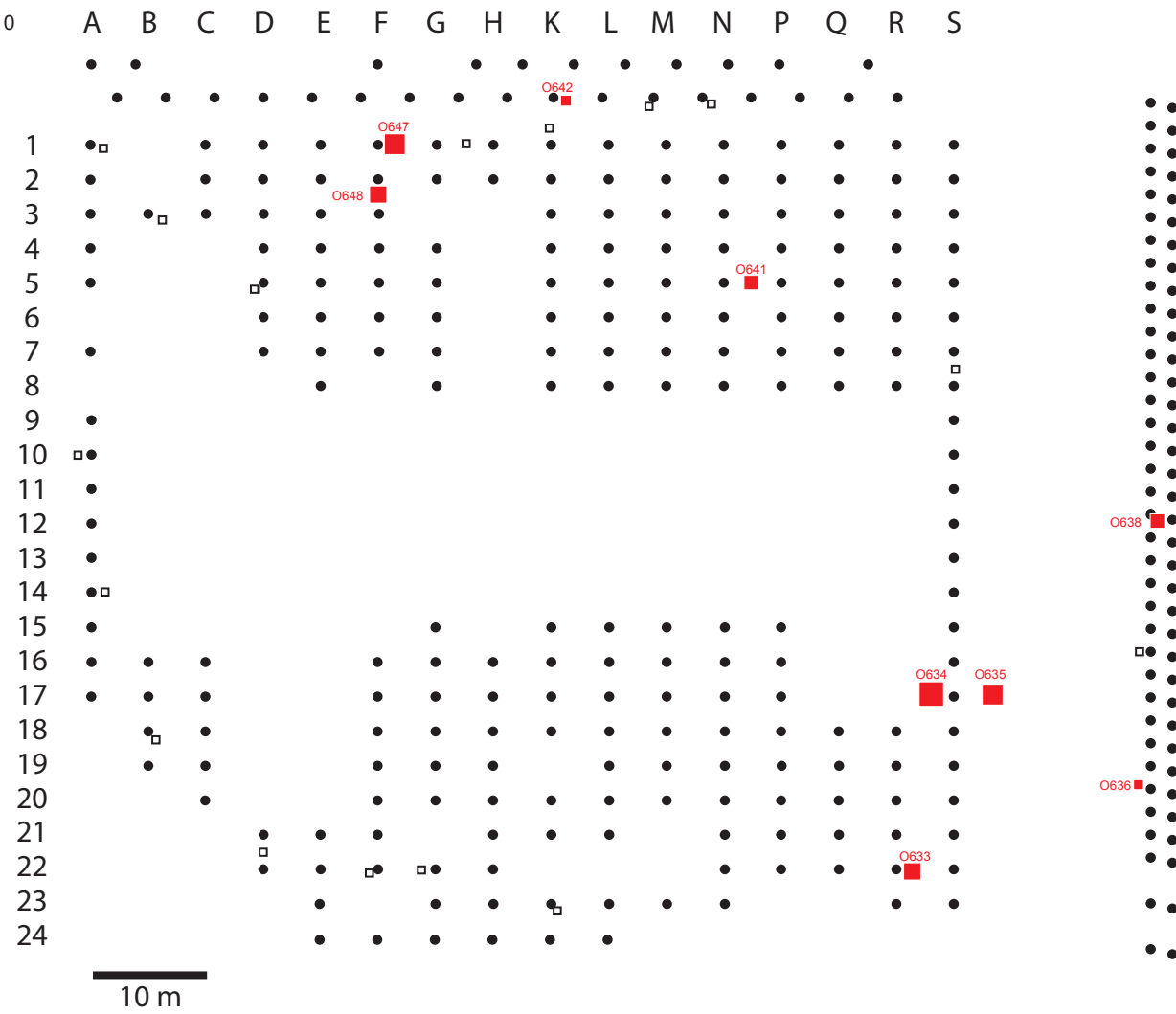

13 Sep 2010  
n=25

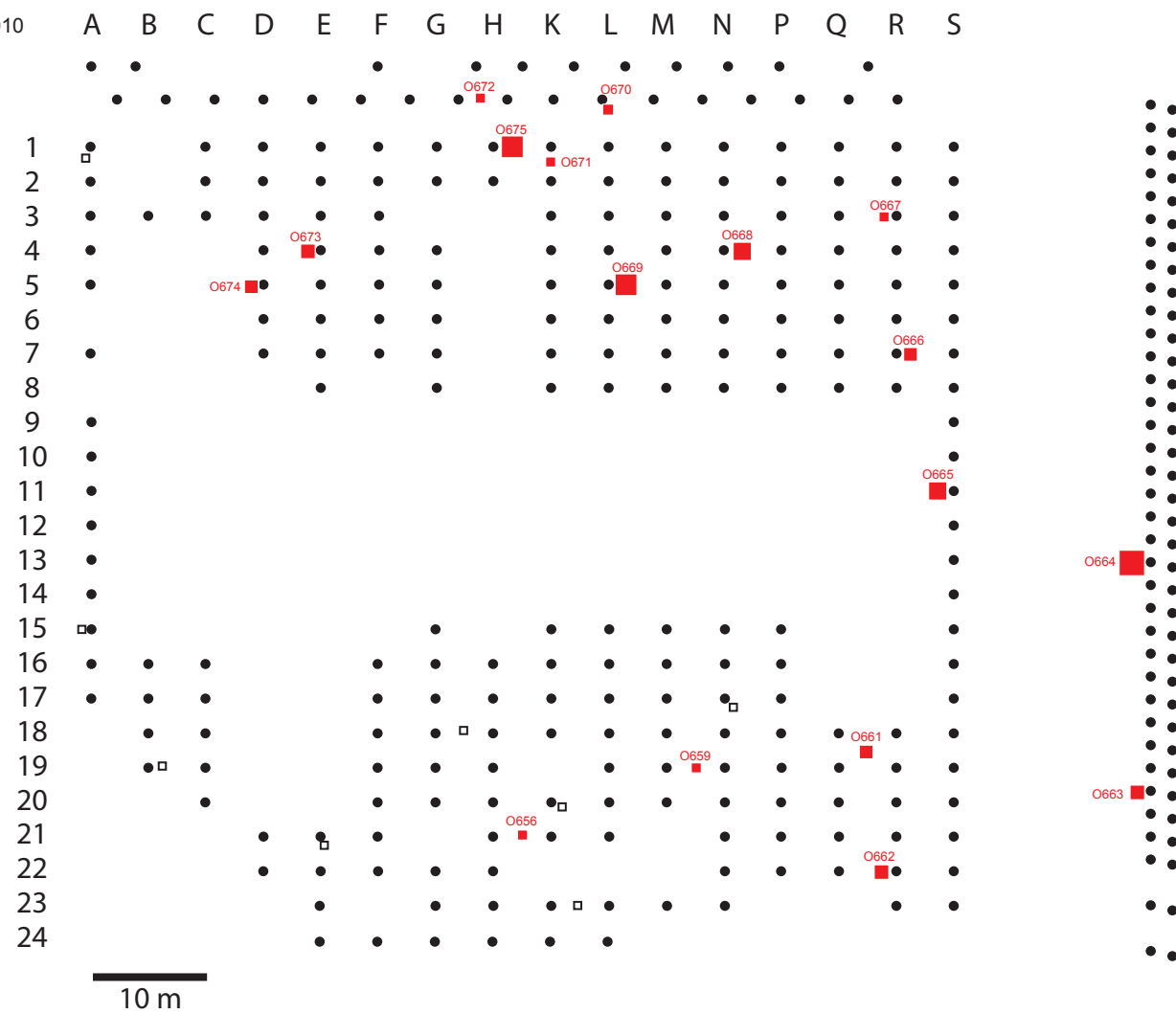

11 Oct 2010  
n=25

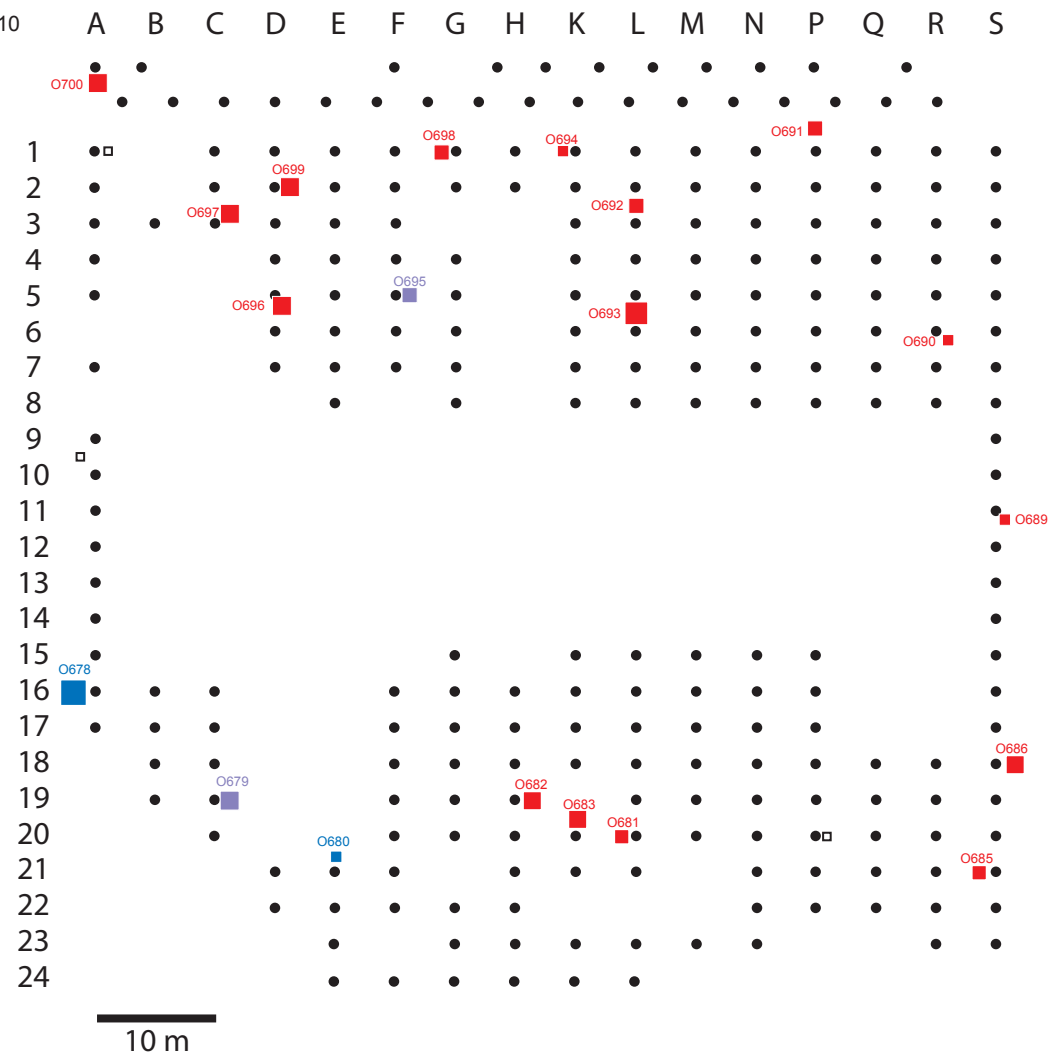

8 Nov 2010  
n=28

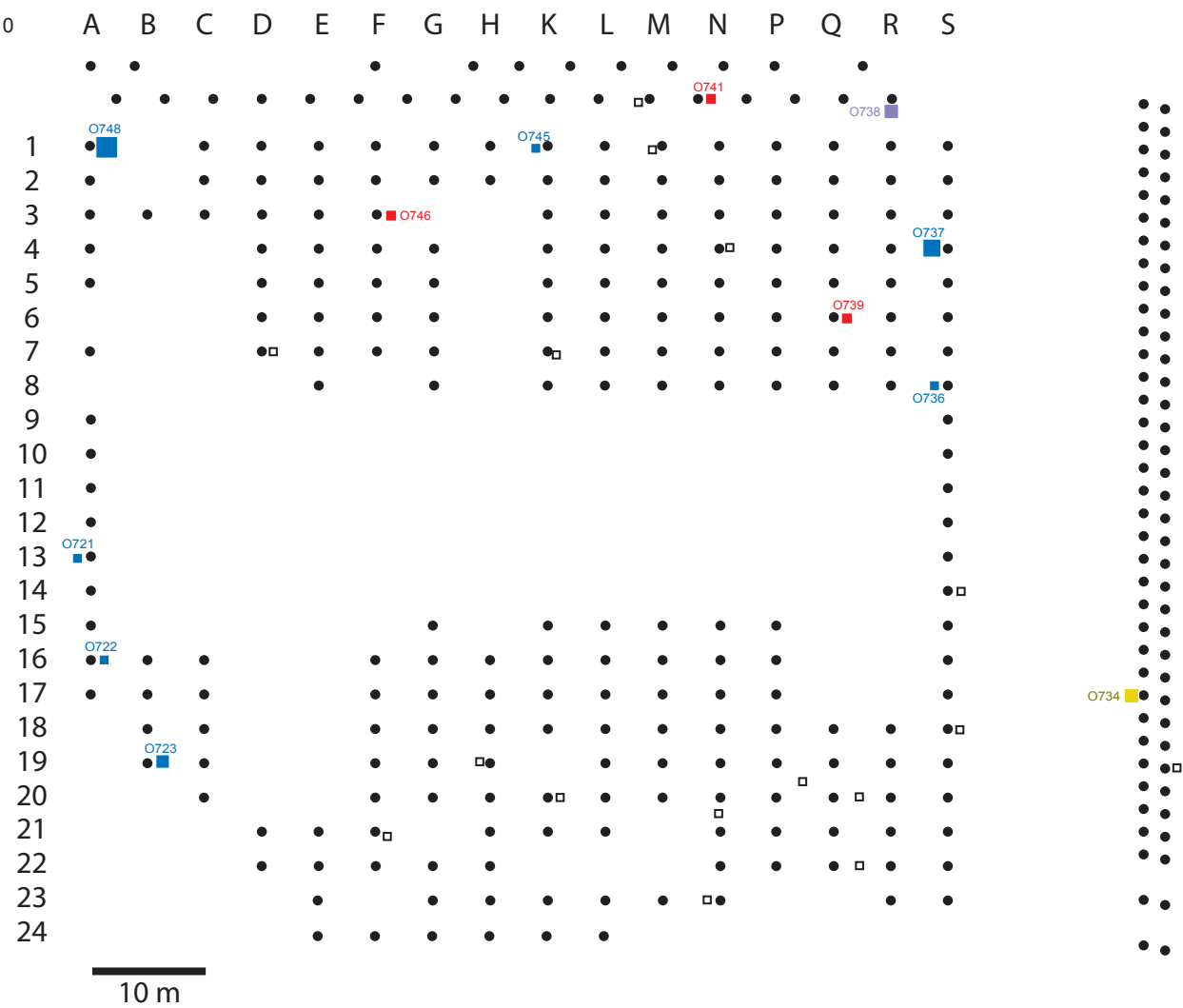

6 Dec 2010  
n=27

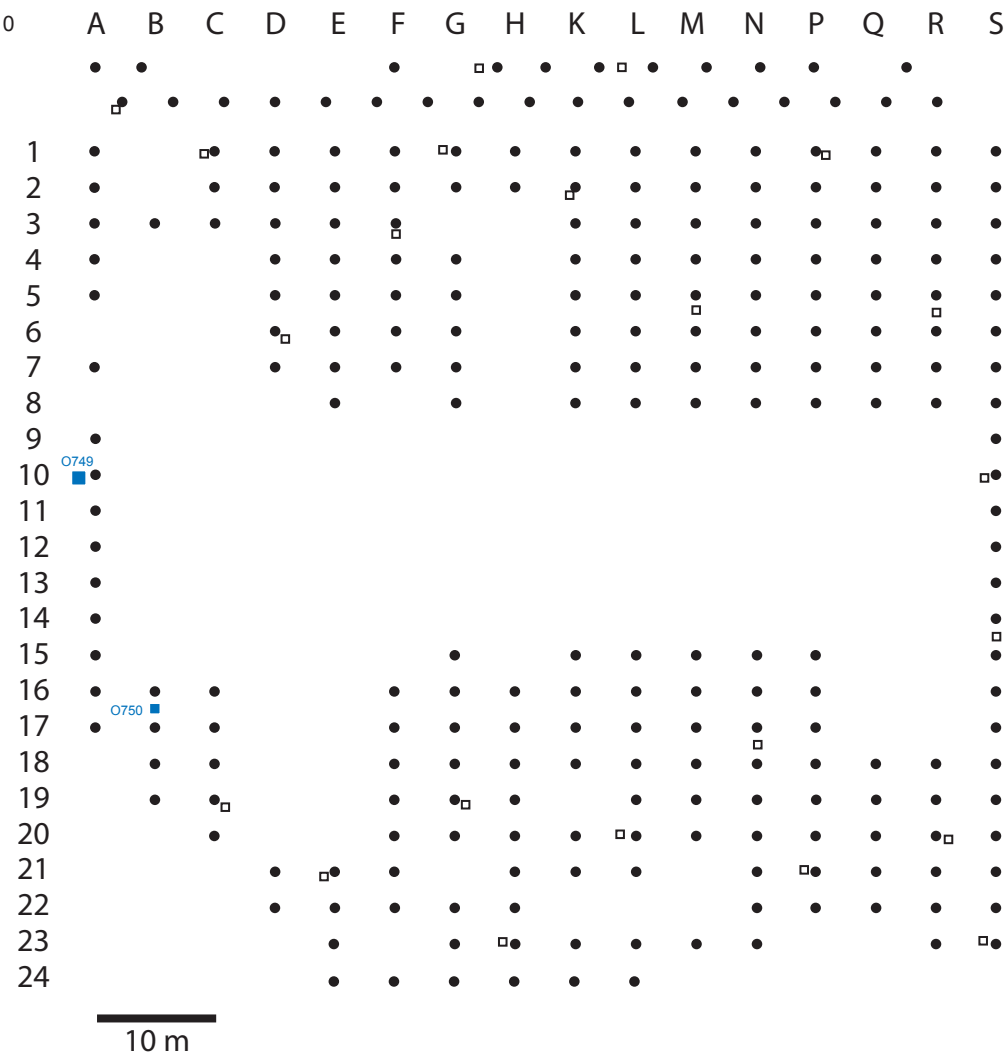

31 Jan 2011  
n=25

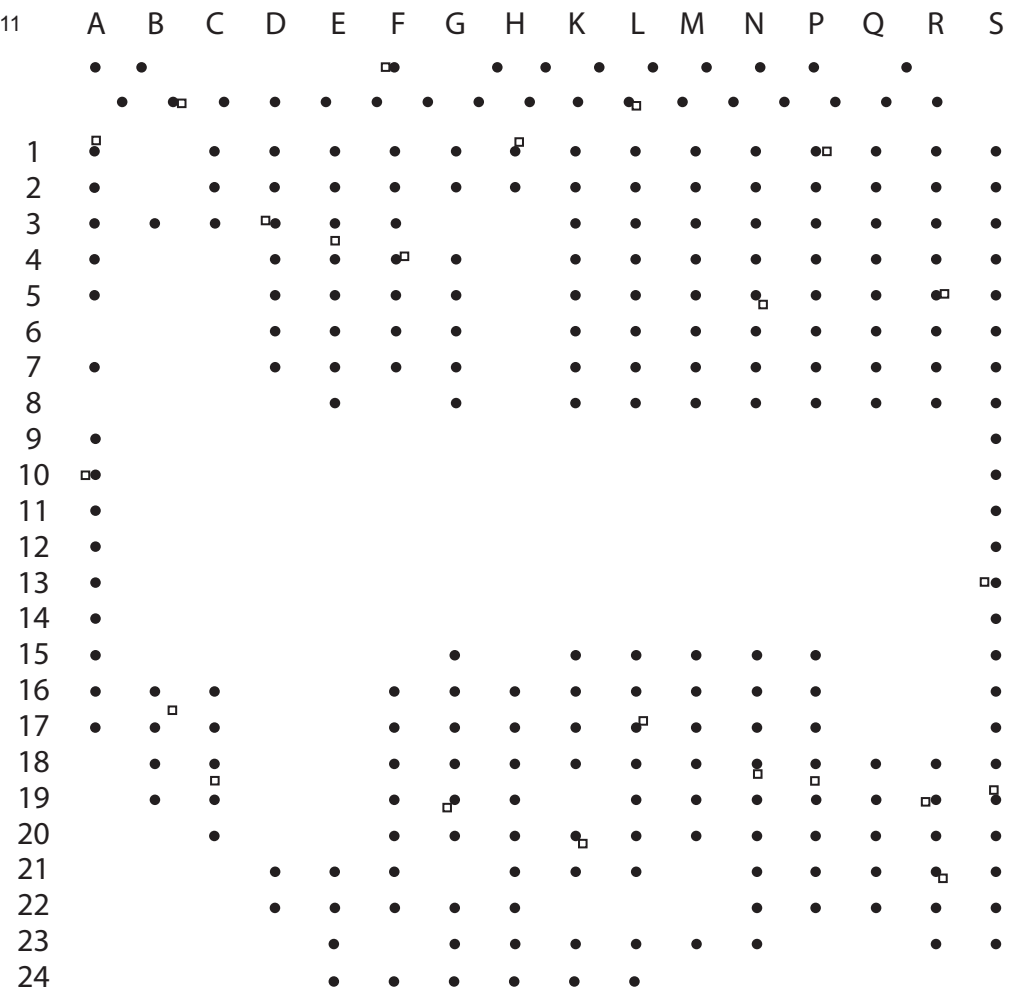

10 m

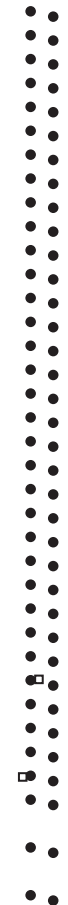

10 Oct 2011  
n=25

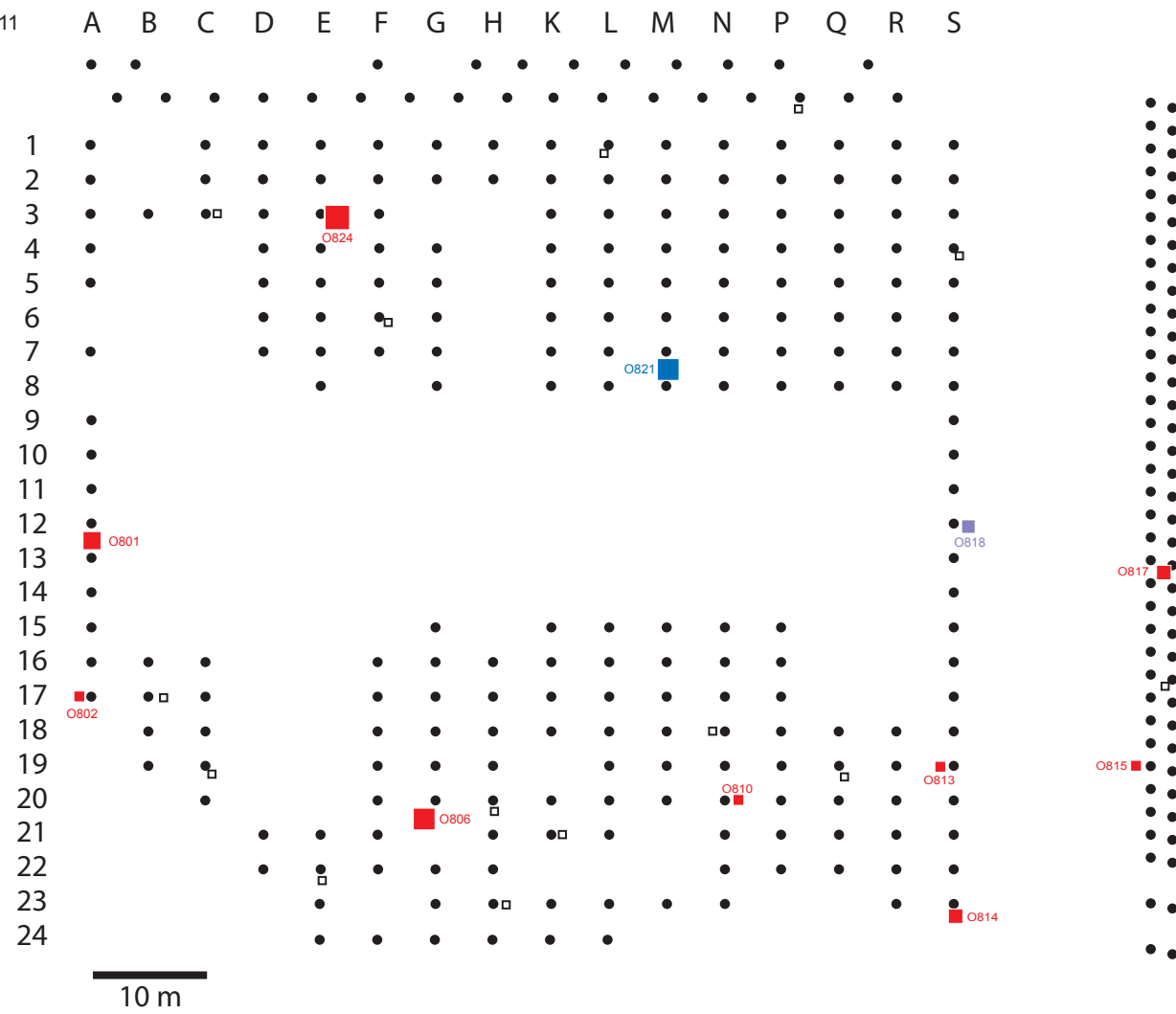

11 Nov 2011  
n=20

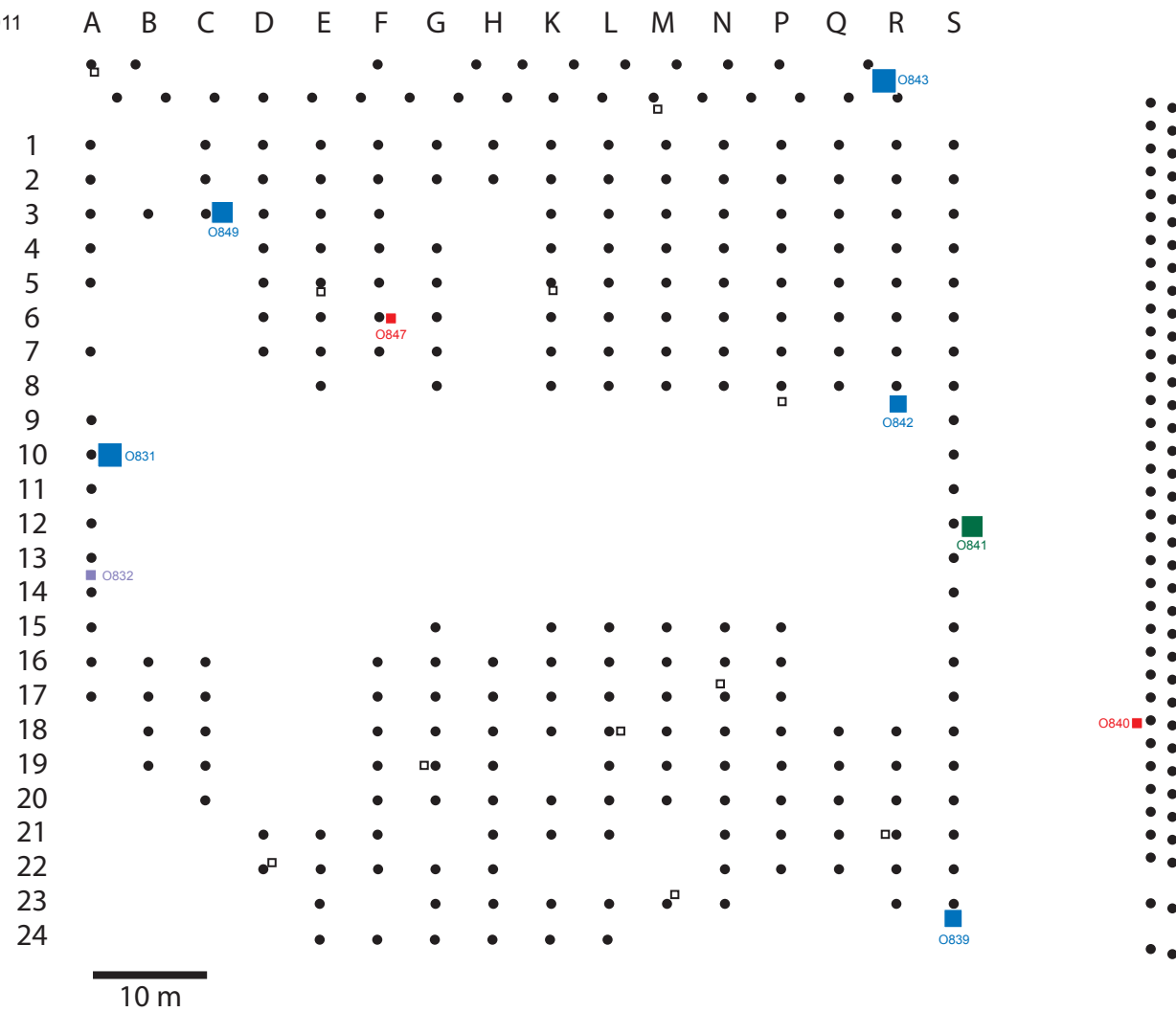

Supplement: Additional file 3 — Spatial distribution of Caenorhabditis species in the Orsay orchard over 19 time points. Black dots: apple trees. Apple positions were noted using the tree(s) they were closest to. Empty square: apple with no Caenorhabditis. The apples with Caenorhabditis are indicated with their sample name, for example "O11", and color-coded according to the species that was/were found. Red: C. briggsae. Blue: C. elegans. Mauve: C. briggsae and C. elegans. Yellow: C. sp. 13. Orange: C. briggsae and C. sp. 13. Green: C. elegans and C. sp. 13. The size of the square is proportional to the Caenorhabditis population size. Representative for each population size are 1 to 10 individuals: apple O14; 11 to 100: apple O13; 102 to 103: apple O15; 103 to 104: apple O145; > 104: apple O535. The sampling date is indicated at the top left of each map; n: number of sampled apples [see Additional File 1 for scoring of these apples]. North is to the top. [file 1741-7007-10-59-S3.PDF]

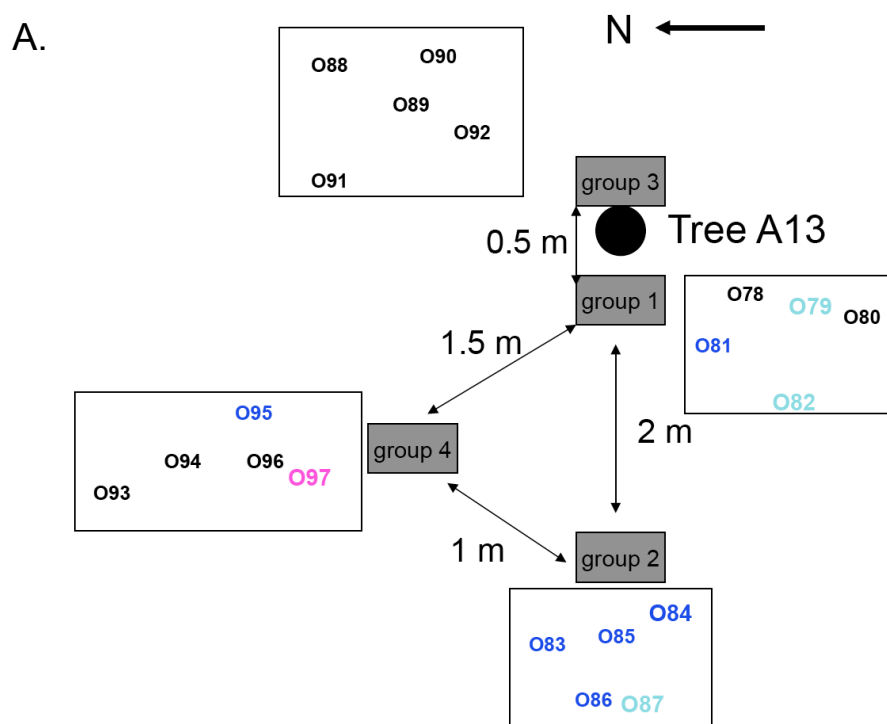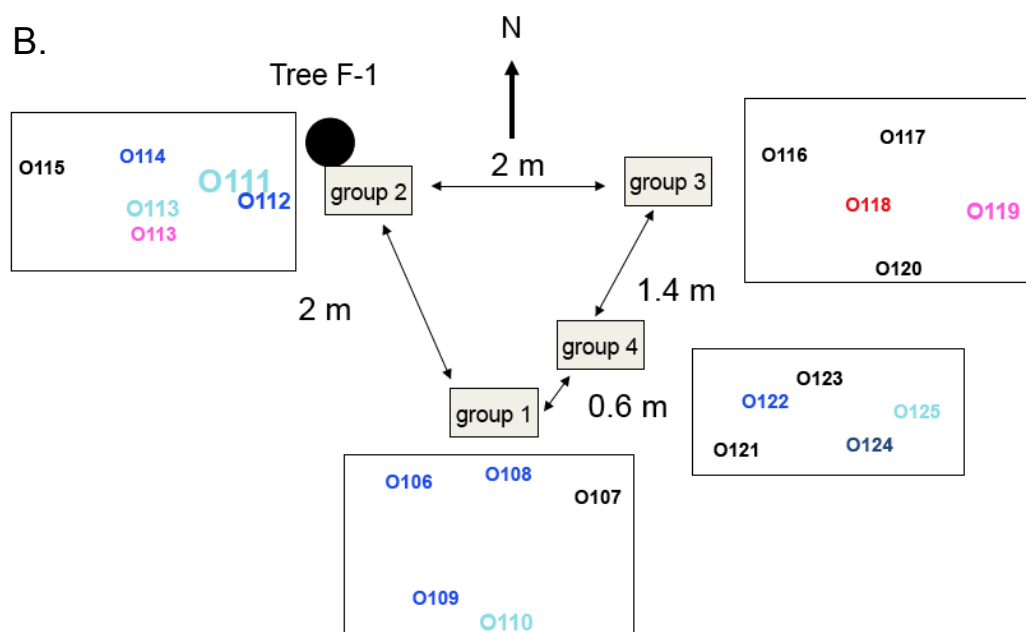

Supplement: Additional file 4 — Spatial distribution of Caenorhabditis species at the scale of a few meters. Twenty apples in four groups of five apples were sampled below a tree on (A) 6 October 2008 and (B) 14 October 2008. The tree trunk is labeled with a black circle. The relative position of each group is shown and the detailed position of apples within a group (within 50 cm of each other) is shown in a close-up in the adjacent rectangle. Blue: apple with C. elegans, with light blue for proliferating populations. Red: C. briggsae, with pink for proliferating populations. Large font size denotes large population size [see Additional File 1 for data]. [file 1741-7007-10-59-S4.PDF]
