# Supplementary material for: Population dynamics and habitat sharing of natural populations of Caenorhabditis elegans and C. briggsae
Source: BMC Biol. 2012 Jun 25;10:59. doi: 10.1186/1741-7007-10-59 (PMC3414772; doi:10.1186/1741-7007-10-59)
Supplement: Additional file 5 — Minimal generation time at 15°C, 21°C and 27°C of the four Caenorhabditis strains used in the competition assays. The minimal generation time (or age at sexual maturity) was estimated from the observation of N = 5 hermaphrodites for each of the four wild isolates grown at the different temperature conditions. JU1530, JU1918, JU1529 and JU2138 are respectively C. elegans isolates from Orsay and Santeuil and C. briggsae isolates from Orsay and Santeuil. Error bars indicate the standard error (SE) of the mean over individuals. A two-way ANOVA (indicated below the graph) was performed to analyze the effect on the age at maturity of the main variables species and temperature, the effect of strain nested in species and the species × temperature interaction term. The box-Cox transformation was applied to the dataset to meet the assumptions of homogeneity of variances. We next performed post-hoc Tukey's HSD test on strain to determine groups of statistical significance. Two bars are significantly different (P < 0.05) if they are not labeled with a same letter. Within each species, the strain genotype has no significant effect on age at maturity at any temperature. However, the two C. elegans strains present a lower age at maturity than the two C. briggsae strains at 15°C, but a higher age at maturity at 21°C and at 27°C. As expected, for both species the minimal generation time decreases when temperature increases. [file 1741-7007-10-59-S5.PDF]

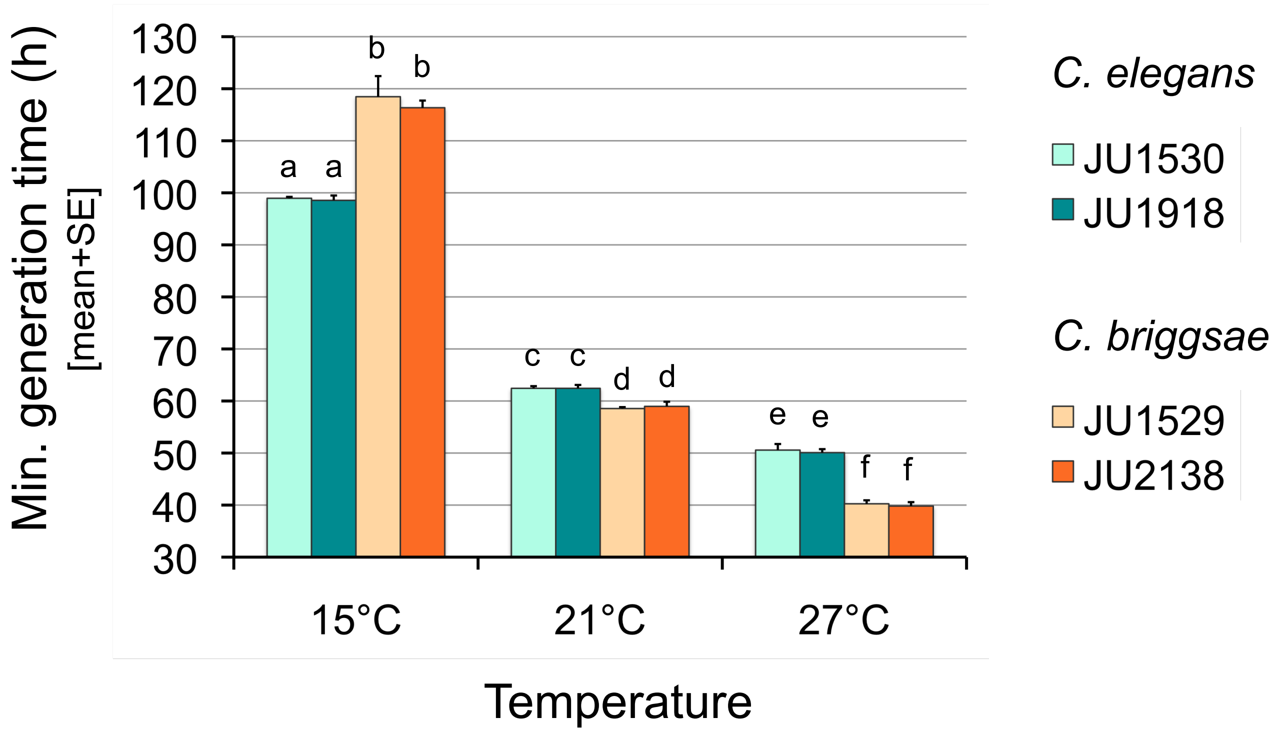

Analysis of variance table.

Source	DF	Sum of squares	Mean square	F value	p value
species	1	0.00573	0.00573	22.1555	p<0.0001
temperature	2	1.97670	0.98853	3819.1472	p<0.0001
strain nested in *species*	2	0.00013	0.00006	0.2443	p=0.78
species x temperature	2	0.10126	0.05063	195.6348	p<0.0001
residuals	48	0.01242	0.00026		

Additional File 5
